# 1,3-Dioxolane derived nucleos(t)ides: synthetic strategies, current position, and future promise in antiviral and anticancer drug discovery

**DOI:** 10.1007/s00044-026-03555-0

**Published:** 2026-04-11

**Authors:** Meghana Gudi, Jeremy Estes, Yugandhar Kothapalli, Stefan G. Sarafianos, Uma S. Singh

**Affiliations:** 1 Innovations in Drug Discovery (IDD), Department of Pharmaceutical and Biomedical Sciences, College of Pharmacy, University of Georgia, Athens, GA USA; 2 Center for ViroScience and Cure, Department of Pediatrics, Emory University School of Medicine, Atlanta, GA USA

**Keywords:** Dioxolane-derived nucleos(t)ides (DDNs), Antivirals, Anticancer, Drug Discovery

## Abstract

To date, altered nucleos(t)ides offer significant potential in antiviral and anticancer drug discovery. In the past several decades, numerous modified nucleos(t)ides have received Food and Drug Administration (FDA) approval for the treatment of viral infections and several cancers. Modifying the base or sugar of a nucleos(t)ide confers specificity and selectivity for viral RNA/DNA polymerases and highly dividing cancer cells. Ribose- and carbocyclic-based nucleosides are well established in medicinal chemistry, yielding many preclinical leads and clinical candidates with therapeutic potential. The insertion of a 1,3-dioxolane ring in place of the ribose sugar represents a unique strategy in nucleoside design to target viral DNA polymerases and rapidly proliferating cancer cells. To date, only a few 1,3-dioxolane-derived nucleos(t)ides (DDNs) have been progressed for clinical development. The chemical space of DDNs remains insufficiently explored. Substantial efforts are still required to develop novel molecules within this class, which may open new avenues for the discovery of potent antiviral and anticancer agents. This review is focused on the synthesis and development of selective DDNs and their potential in preclinical/clinical development.

**Figure:**
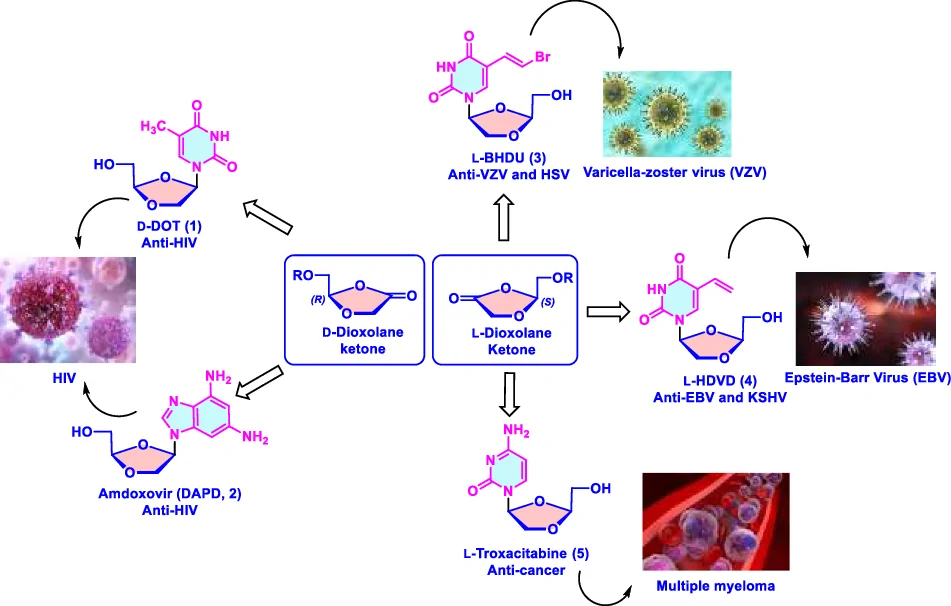

## Introduction

**Fig. 1:**
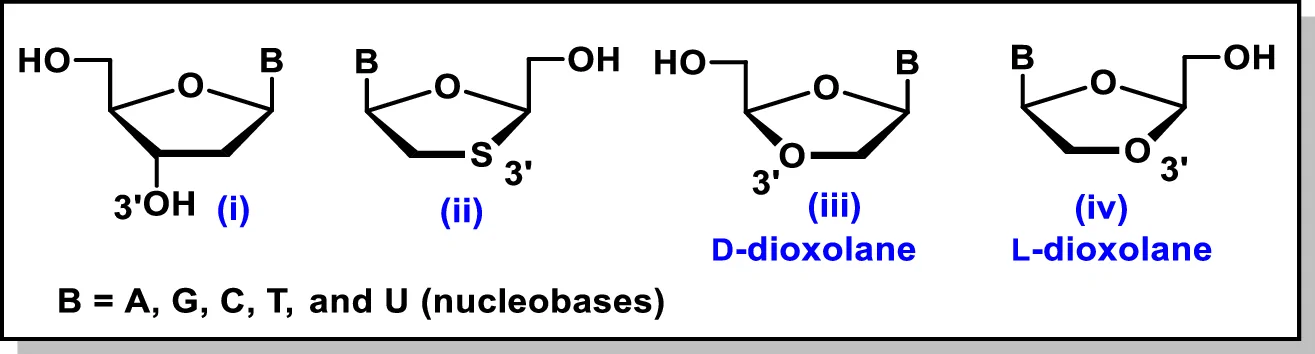
Representation of (i) D-2ʹ-deoxyribose, (ii) L-oxathiolane, (iii) D-dioxolane, (iv) L-dioxolane, derived nucleoside analogs. Compared to the 2ʹ-deoxyribose nucleosides, 3′-OH is absent in both oxathiolane- and dioxolane-derived nucleosides. B represents nucleobase A adenine, G guanine, C cytosine, U uracil; and T thymine

1,3-Dioxolanes, heterocyclic acetal cyclopentyl rings, offer a potential replacement of the sugar scaffold in nucleosides to construct either D- or L-dioxolane-derived nucleos(t)ides (DDNs) [1, 2]. These molecules both with D- (*natural*) and L- (*unnatural*) configuration lack the crucial 3ʹ-OH necessary to propagate DNA chain elongation (Fig. 1). Additionally, replacing a ribose or 2ʹ-deoxy-ribose sugar with a dioxolane ring in nucleosides, generates specificity and selectivity for viral polymerases without causing significant cytotoxicity to human cells. DDNs may act as both viral polymerase inhibitors as well as viral DNA chain terminators. Mechanistically, the incorporation of either D- or L- dioxolane-derived nucleotide into an elongating viral DNA chain ends up with chain termination, which ultimately inhibits viral growth [3–5]. Oxathiolanes [Fig. 1(ii)], similar to dioxolanes, though with a 3′ sulfur in lieu of an oxygen, also have potent antiviral activity [6]. Lamivudine (3TC) and emtricitabine (FTC), derived from oxathiolane sugars, are FDA-approved drugs for the treatment of HBV and HIV infections, respectively [6].

**Fig. 2:**
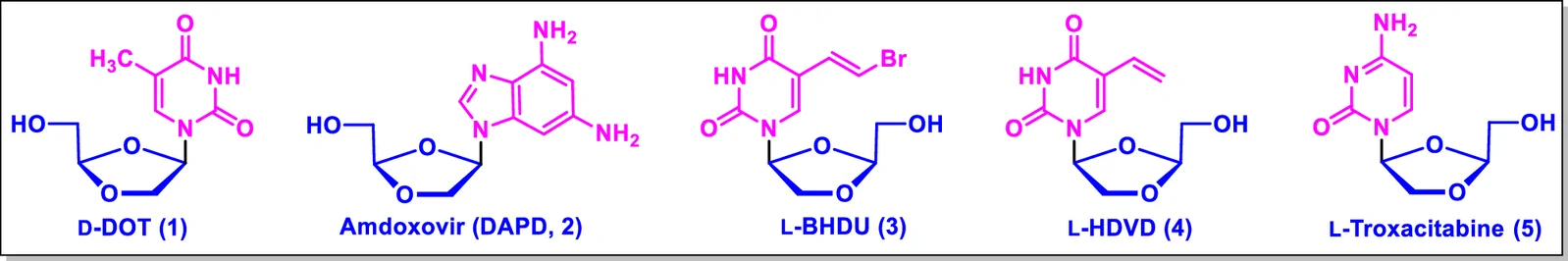
Structures of potent D- and L-dioxolane-derived nucleoside analogs

In the past, dioxolane nucleos(t)ides have shown potent antiviral and anticancer activity. Our antiviral drug discovery group in the College of Pharmacy at the University of Georgia, and several others have previously synthesized numerous dioxolane analogs; some of these analogs have shown promising antiviral and anticancer activity [1, 7–13]. D-DOT (**1**, Fig. 2), a dioxolane thymidine nucleoside, and its prodrugs have exhibited potent anti-HIV activity [14]. Amdoxovir (DAPD, **2**), a diaminopurine dioxolane nucleoside, is a potent HIV replication inhibitor [3]. Bromovinyl uracil L-BHDU (**3**) and vinyl uracil L-HDVD (**4**) are dioxolane nucleosides with strong activity against herpesviruses [4, 15]. L-BHDU showed activity primarily against varicella-zoster virus (VZV) [15], whereas L-HDVD exhibited broad-spectrum antiviral activity against Epstein–Barr virus (EBV), Kaposi’s sarcoma-associated herpesvirus (KSHV), and herpes simplex virus 1 (HSV-1) [4]. Troxacitabine (**5**), an L-dioxolane pyrimidine analog, has shown promising anticancer activities across numerous cancers, and has also exhibited anti-HIV and anti-HBV activity[2, 16, 17]. Among these molecules, DAPD and troxacitabine advanced into clinical development. Currently, POM-L-BHDU-MP [7,18–20] (a phosphate ester prodrug of L-BHDU) as well as L-HDVD [4] are under active investigation in preclinical and clinical studies for the treatment of herpesvirus infections.

**Fig. 3:**
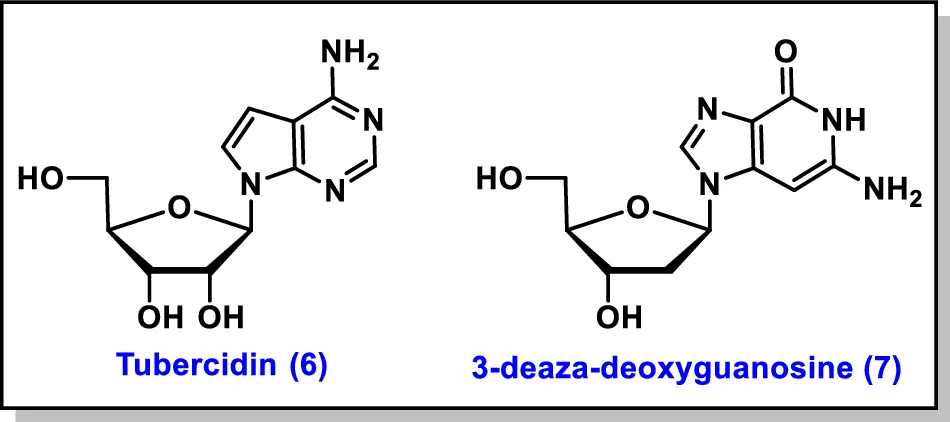
Structures of deazapurine nucleosides with potent antiviral activity

There remains a significant need to develop highly efficacious and selective dioxolane nucleosides as antiviral agents. Achieving this goal will likely require additional structural alterations, either on the dioxolane ring or on the nucleobase. Such modifications increase molecule complexity and often confer improved selectivity toward specific viruses. Additionally, replacing nitrogen with carbon in the nucleobase can have a profound effect on antiviral activity. This substitution removes a potential hydrogen-bond acceptor or donor site, which can substantially alter the molecule’s binding properties and overall profile. Inspired by these modified-base strategies, 7-deazapurines and other altered-base nucleos(t)ides have attracted considerable attention as promising antiviral agents [2]. For example, tubercidin (**6**, Fig. 3), a 7-deazaadenosine analog, and its derivatives exhibit nanomolar potency against hepatitis C virus (HCV), as well as notable anticancer activity [21, 22]. 3-Deaza-2ʹ-deoxyguanosine (**7**), another deazapurine analog, demonstrated antiviral activity against both RNA and DNA viruses [2].

Furthermore, the strategic insertion of a D-dioxolane ring in combination with a 2,6-diaminopurine base led to the development of amdoxovir (**2**) [8]. Subsequent replacement of the purine with a pyrimidine base yielded DOT (**1**), which exhibited potent anti-HIV activity [13]. The incorporation of a L-dioxolane with cytosine base produced troxacitabine (**5**), a highly effective anticancer agent [23]. These examples illustrate how structural modifications at the 2ʹ- and 4ʹ-positions of DDNs, together with changes to the nitrogenous purine and pyrimidine bases, can open promising new pathways for developing novel antiviral and anticancer agents. Although significant medicinal chemistry efforts are currently underway, further research is still needed. This review focuses on the synthesis, biological activity, preclinical studies, and clinical development of selected DDNs (Fig. 2). It aims to provide a valuable resource for medicinal chemists, virologists, and chemical biologists interested in revisiting these analogs and exploring their therapeutic potential in the antiviral and anticancer fields.

**Figure:**
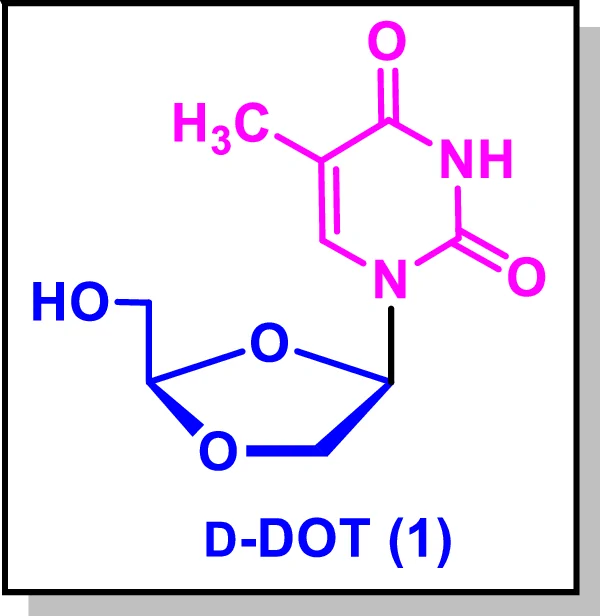

### D-DOT (1)

The chemical name of D-DOT is (-)-*β*-D-(2 *R*,4 *R*)-dioxolanylthymine, and it is a thymidine-based nucleoside analogue. D-DOT features a D-dioxolane ring in place of the furanose ring, forming a distinctive dioxolane-cyclopentyl-like structure. D-DOT has shown promising antiviral activity against HIV-1 while exhibiting no significant cytotoxicity in preclinical studies [24, 25]. The antiviral potency of D-DOT as a nucleoside reverse transcriptase inhibitor (NRTI) was assessed through a series of biochemical assays, where its inhibitory activity was compared with that of clinically approved NRTIs [25]. In initial cell-based studies, D-DOT showed promising anti-HIV-1 activity in the ATH8 cell line and human peripheral blood mononuclear cells (PBMC), with notably lower cytotoxicity than the standard drug azidothymidine (zidovudine, AZT) [16, 26]. Following these encouraging initial results, D-DOT was advanced as a preclinical candidate. To support further biological evaluation of D-DOT, several synthetic approaches were developed. In parallel, various prodrug strategies were explored to improve its antiviral profile. These included amino acid esters [13], short- and long-chain lipid phosphate derivatives [13], and phosphoramidate prodrugs [27], all of which were synthesized and tested for antiviral activity. Although none of these prodrugs demonstrated superior antiviral potency than the parent nucleoside (D-DOT), the modifications may still offer advantages in pharmacokinetic (PK) properties of D-DOT, including improved oral bioavailability and enhanced cellular uptake.

**Scheme 1:**
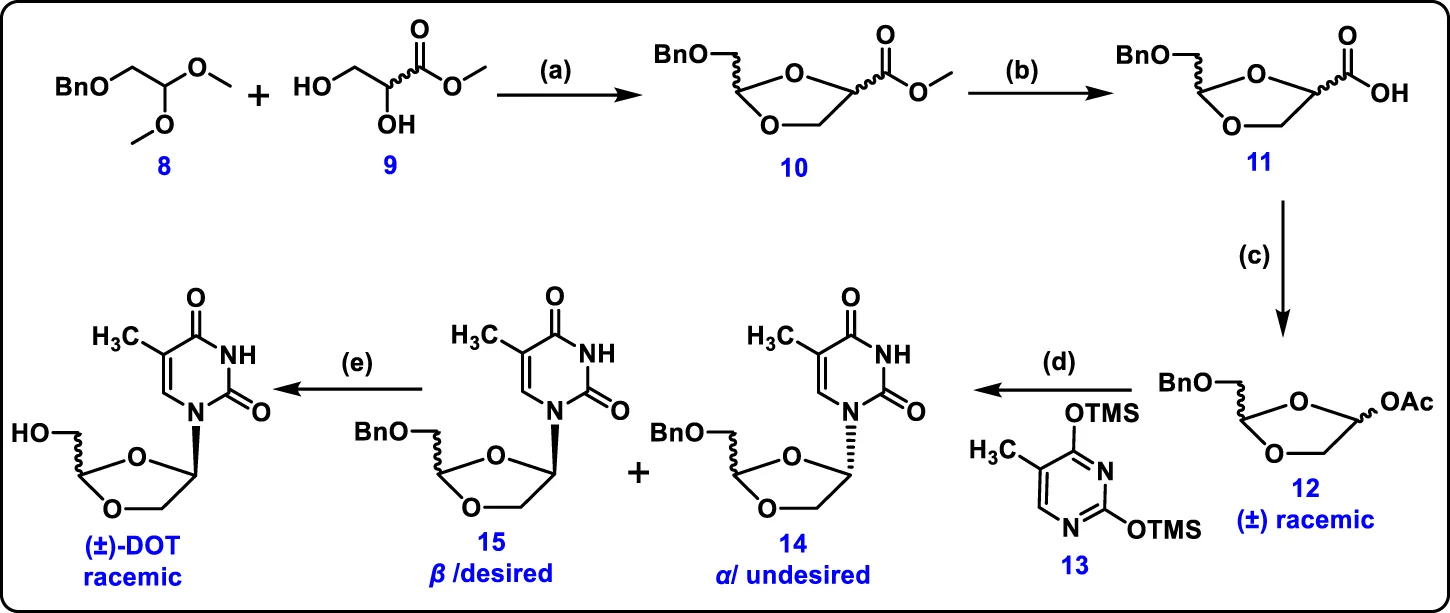
Initial synthesis of racemic (±)-DOT. *Reagents and conditions: (*a) *p*-TsOH, CH_3_CN; (b) LiOH, THF/H_2_O; (c) Pb(OAc)_4_, pyridine, CH_3_CN; (d) TMSOTf, DCM; (e) Pd(OH)_2_/C, H_2_, EtOH

As depicted in Scheme 1, first, the synthesis of racemic (±)-DOT was achieved by Norbeck et al [28]. The synthesis of (±)-DOT (mixture of both L- and D- DOT) began with the condensation of 1-benzyloxy-2,2-dimethoxyethane **8** with methyl glycerate **9**, yielding the diastereomeric dioxolane ester intermediate **10**. Saponification of the methyl ester **10** was carried out by using aqueous lithium hydroxide (LiOH) in a 4:1 THF/water mixture to produce the carboxylic acid intermediate **11**. Furthermore, oxidative decarboxylation of **11** was performed in the presence of lead (IV) acetate [Pb(OAc)_4_] to generate crucial acylated racemic intermediate **12**.

Finally, racemic (±) dioxolane acetate **12** was coupled with trimethylsilyl thymine **13** under Vorbrüggen coupling conditions in the presence of trimethyl trifluoromethanesulfonate (TMSOTf) to produce racemic α/undesired **14**, and *β*/desired **15** protected nucleosides, which were separated by silica gel column chromatography to obtain desired *β* isomer **15** in good yield. Finally, nucleoside **15** was treated with palladium hydroxide [Pd(OH)_2_] on carbon under a hydrogen atmosphere to produce the final 1:1 racemic (±)-DOT.

**Scheme 2:**
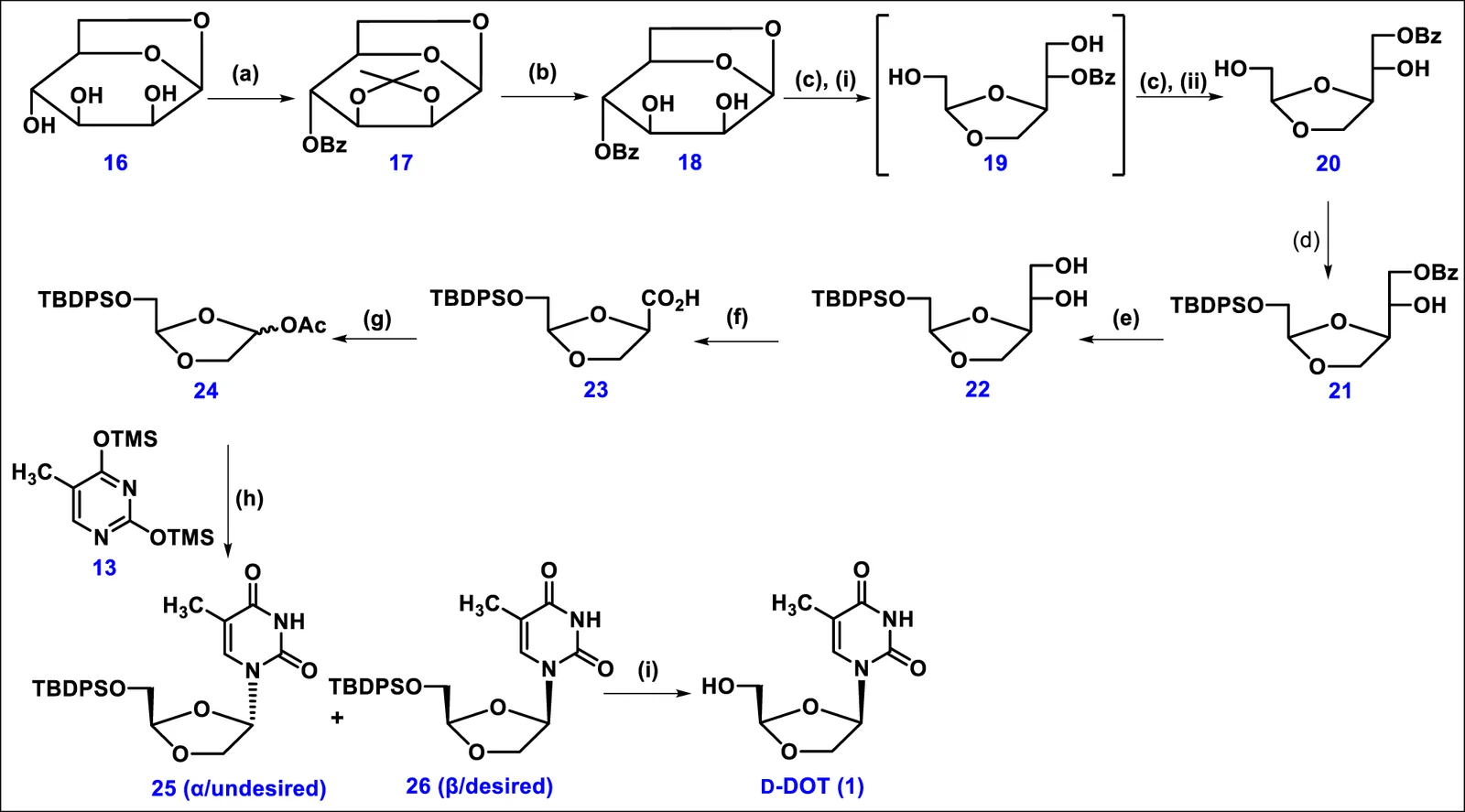
Improved synthesis of chiral pure D-DOT from 1,6-anhydro-D-mannose **16**. *Reagents and conditions*: (a) i) Acetone, H^+^; ii) BzCl, pyridine; (b) H_2_SO_4_, 60% dioxane/H_2_O; (c) i) NaIO_4_, 1:1 H_2_O/EtOH; ii) NaBH_4_, MeOH; (d) Base catalyzed benzoyl migration;[26] (e) i) TBDPSCl, imidazole, DMF; ii) NaOMe, MeOH; (f) NaIO_4_/RuO_2,_ CCl_4_/ACN/H_2_O (1:1:1.5); (g) Pb(OAc)_4,_ EtOAc; (h) TMSOTf, DCM; (i) TBAF, THF

Chu et al optimized the asymmetric enantiomeric synthesis of chiral D-DOT [26]. To retain the absolute stereochemistry of the chiral D-dioxolane key intermediate **24** [16, 26], the synthesis was initiated with 1,6-anhydro-D-mannose **16** (Scheme 2). The 2,3-dihydroxy group of **16** was protected with an isopropylidene group. Compound **16** was treated with acetone in an acidic medium to give a 2,3-isopropylidene-protected intermediate, which, upon further treatment with benzoyl chloride in pyridine, produced fully protected compound **17**. Next, the isopropylidene group of **17** was deprotected with diluted sulfuric acid to afford intermediate **18**. The vicinal diol groups of **18** were oxidized with sodium periodate (NaIO_4_)_,_ followed by reduction with sodium borohydride (NaBH_4_) to produce intermediate **19**.

The intermediate **20** is formed spontaneously *via* benzoyl migration of **19** under a base-catalyzed condition [26]. Furthermore, the primary hydroxy group of **20** was treated with *tert*-butyldiphenylsilane chloride (TBDPSCl) in the presence of imidazole in DMF to form a TBDPS-protected intermediate (**21**), which was treated with sodium methoxide in methanol to furnish the benzoyl deprotected terminal diol compound **22**. Oxidation of intermediate **22** was carried out by NaIO_4_/ ruthenium(IV) oxide (RuO_2_) in a mixture of carbon tetrachloride (CCl_4_), acetonitrile (ACN), and water (1:1:1.5) to produce intermediate **23**. The carboxylic acid of **23** was treated with Pb(OAc)_4_ in ethyl acetate (EtOAc) to furnish diastereomeric acetate key intermediate **24** in ratio of *α*/*β* 2:1. Finally, the condensation of intermediate **24** with silylated thymidine **13** in the presence of TMSOTf in DCM was carried out to construct coupled products as *α*-undesired isomer **25** and *β*-desired isomer **26**. The TBDPS deprotection of **26** was carried out using tetrabutylammonium fluoride (TBAF). Compound **26** was treated with a 1 M solution of TBAF in THF to give the final chiral compound D-DOT (**1**).

**Scheme 3:**
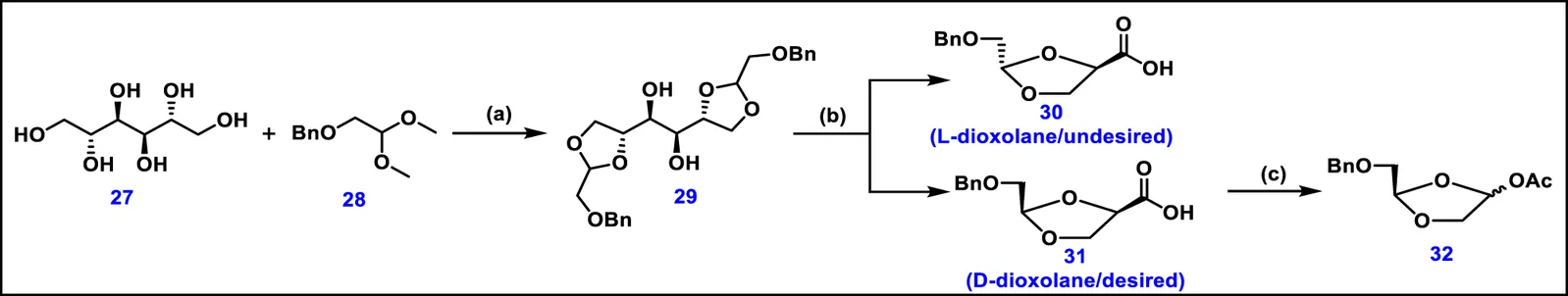
Modified synthesis of chiral pure D-intermediates (**32**). *Reagents and conditions:* (a) SnCl_2_, DME; (b) RuCl_3_ hydrate, NaOCl, in H_2_O:ACN:DCE 2:1.4:6; (c) Pb(OAc)_4_, ACN, DCM, pyridine

Later, Evans et al explored alternative protocols to obtain chiral pure D-DOT and L-DOT by synthesizing and separating chiral intermediates **30** and **31** (Scheme 3) [29]. The synthesis of chiral pure **30** and **31** began by protecting the 1,2- and 5,6-diol moieties of D-mannitol (**27**). Compound **27** was treated with benzyloxyacetaldehyde dimethylacetal **28** in the presence of SnCl_2_ in DME to produce protected intermediate **29**.

**Scheme 4:**
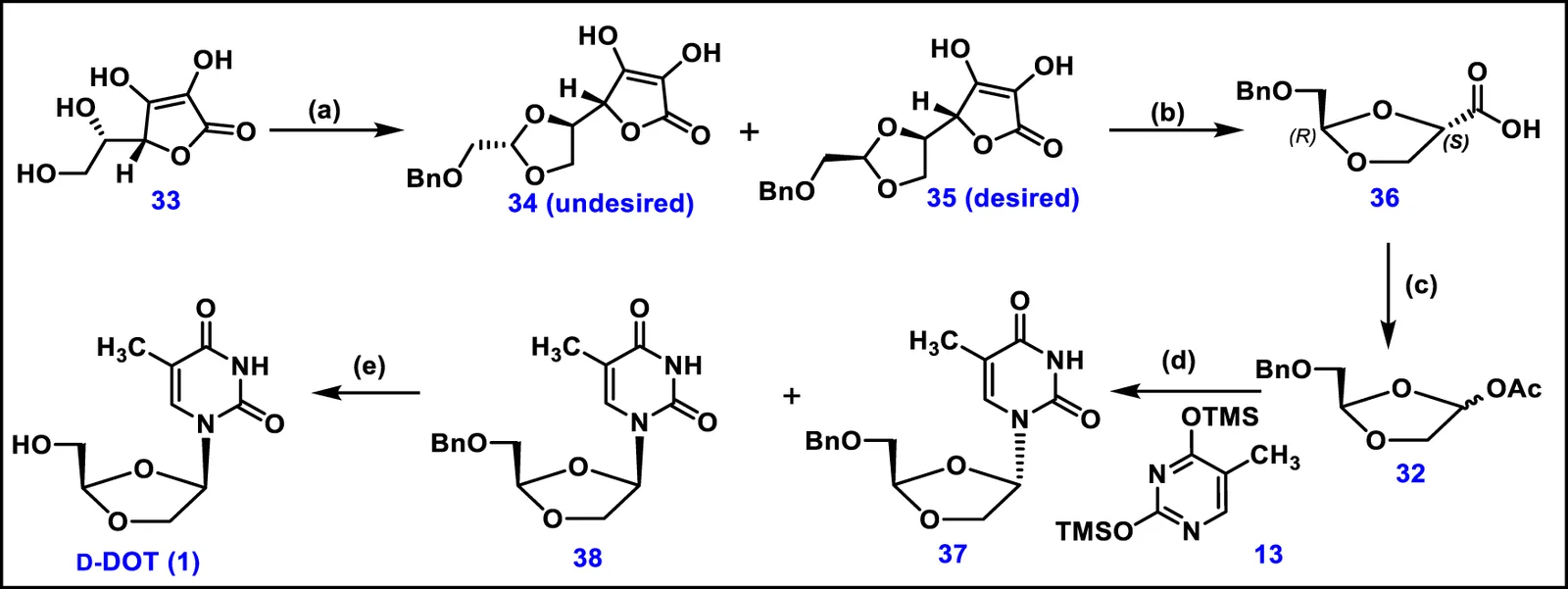
Revised efficient synthesis of chiral D-DOT *via* intermediate **32**. *Reagents and conditions*: (a) (2,2-dimethoxyethoxy)methyl)benzene, TsOH/ACN; (b) i) 30% H_2_O_2_, K_2_CO_3_, EtOH; ii) RuCl_3_ hydrate, NaOCl, DCE-CH_3_CN-H_2_O, BnEt_3_NCl, pH 8, EtOH; (c) Pb(OAc)_4_, CH_3_CN-DCM-pyridine; (d) TMSOTf, DCM; (e) 10% PdO, cyclohexene, EtOH

Next, oxidative cleavage of 3,4-diols of intermediate **29** was performed by RuCl_3_ and NaOCl in a mixture of solvent water:ACN and DCE in a ratio of 2:1.4:6 to yield carboxylic acids of chiral pure L-dioxolane **30** and D-dioxolane **31**. Furthermore, **31** was converted to crucial acetate key intermediate **32**. Compound **31** was treated with Pb(OAc)_4_ to afford **32**. However, during the synthetic development of intermediate **32**, Evan’s group redirected their efforts toward Scheme 4, which proved to be a more efficient and practical approach for the construction of crucial coupling intermediate **32**. First, the 5,6-diol moiety of L-ascorbic acid **33** was protected with (2,2-dimethoxyethoxy)methyl)benzene to furnish a diastereomeric mixture of compounds **34** and **35** (Scheme 4). Compound **33** was condensed with (2,2-dimethoxyethoxy)methyl)benzene in the presence of catalytic *p*-toluenesulfonic acid (*p*-TsOH) in ACN affording a diastereomeric mixture of protected derivatives **34** and **35**.

The desired isomer **35** was selected for further conversion. Compound **35** was then subjected to oxidative cleavage: treatment with hydrogen peroxide (H_2_O_2_) in the presence of K_2_CO_3,_ followed by oxidative treatment of ruthenium chloride hydrate and sodium hypochlorite (NaOCl) furnished the carboxylic acid intermediate **36**. Subsequent decarboxylative oxidation of **36** with Pb(OAc)_4_ under basic conditions (following previously reported procedures) [26, 29] provided the key intermediate **32**. Coupling of silylated thymine **13** with **32** yielded a mixture of *α*- and *β*- nucleoside isomers. The benzyl deprotection of desired *β*-isomer **38** yielded the target compound D-DOT **(1)**.

### Antiviral activity of D-DOT (1)

The racemic (±)-DOT was initially screened against wild-type (WT) HIV-1 in ATH8 cells by measuring cytopathic effect, yielding an EC_50_ of 20 µM (Table 1), compared to 0.5 µM for the reference antiviral AZT [28]. Although racemic (±)-DOT was significantly less potent than AZT, its substantially lower cytotoxicity made it an interesting candidate for further antiviral evaluation. Subsequent testing in PBM cells revealed much greater potency of racemic (±)-DOT, with an EC_50_ of 0.09 µM and no detectable cytotoxicity up to CC_50_ > 100 µM (in Vero cells). In the same assay, AZT showed an EC_50_ of 0.009 µM and racemic (±)-DOT was found to be approximately 10-fold less potent [16]. Since racemic (±)-DOT comprises both D- and L-isomers, a key goal was to identify which isomer was primarily responsible for the antiviral activity, Chu et al re-evaluated the pure chiral D-DOT and revealed its EC_50_ of 0.3 μM in PBM cells as well as in ATH8 cells [16, 26]. Importantly, D-DOT retained strong antiviral activity against the lamivudine-resistant M184V mutant (EC_50_ = 0.088 µM) [16, 30]. It also showed activity against AZT-resistant strains 9 F with EC_50_ values of 4.9 and an EC_50_ of 0.2 µM against AZT-sensitive 10 strain without cytotoxicity up to CC_50_ > 100 µM.

**Table 1:** Antiviral activity of both racemic (±)-DOT and chiral D-DOT

Compound	HIV-1 Type	Cell Lines	EC_50_ (μM)	CC_50_ (µM)	Reference
( ± )-D- DOT	WT^a^	ATH8	20	>200	[28]
		PBMC	0.09	>100	[16]
(-)-D-DOT	WT	“	~ 0.30	>100	[16, 26]
	M184V^b^	“	0.088	>100	[30]
	AZT-resistant 9 F	“	4.9	>100	[16]
	AZT-sensitive 10	“	0.2	>100	
	K65R^b^	“	0.21 ± 0.55	>100	[24]
	L74V^b^	“	0.33 ± 0.93	>100	
	T215Y^b^	“	0.27	>100	
	M184V/Q151M^b^	“	0.23 ± 0.20	>100	
	D67N/K70R/T215Y/K219Q^b^	“	0.49 ± 0.21	>100	
	HIV-1 LAI	“	0.68	>100	[13]

^a^WT: Wild type

^b^systematic HIV-1 RT mutations associated with common drug resistance patterns

Additionally, D-DOT retained its antiviral profile against NRTI-resistant HIV-1 reverse transcriptases (HIV-RT) with K65R (EC_50_ = 0.21 µM), L74V (EC_50_ = 0.33 µM), T215Y (EC_50_ = 0.27 µM), M184V/Q151M (EC_50_ = 0.23 µM) and D67N/K70R/T215Y/K219Q (EC_50_ = 0.49 µM) mutations (Table 1) [24]. In repeated screening against the HIV-1 LAI strain in PBM cells, D-DOT maintained its antiviral potency of EC_50_ = 0.68 µM. Additionally, in Vero cells, D-DOT expressed moderate antiviral activity (EC_50_ = 24.6 μM) against severe acute respiratory syndrome coronavirus (SARS-CoV) without any significant cytotoxicity (CC_50_ > 100 µM) [31].

Molecular modeling studies of D-DOT with wild-type as well as with mutated HIV RT (HIV reverse transcriptase) strains have been examined [24, 30]. Docking studies revealed that D-DOT triphosphate (DOT-TP) maintains a series of hydrogen-bonding interactions with the HIV-1 RT binding site. The bonding between the DOT-TP and adjacent amino acid residues (Lys65, Lys73, Arg72, Tyr115 and/or Gln151) played crucial roles in stabilizing the enzyme-inhibitor complex. Particularly in the case of AZT-resistant mutant, Asp113 and Ala114 demonstrated promising binding affinity to DOT-TP. The dioxolane ring consistently appear to adopt a 3ʹ -exo conformation contrary to the endo conformation adopted by most natural deoxyribonucleoside triphosphates (dNTPs) interacting with HIV RT. The 3ʹ oxygen atom also appeared to express stronger interactions in the binding pocket of mutant HIV RT strains.

Several prodrug strategies have been implemented to improve the antiviral profile of D-DOT. Specifically, the 5ʹ-*O*-aliphatic and amino acid ester prodrugs, including phosphoramidate prodrugs of D-DOT, were synthesized [13]. However, the application of prodrug strategies to D-DOT did not result in a significant enhancement of antiviral activity; nevertheless, such approaches may offer potential advantages in improving the PK profile of D-DOT. In preclinical development, the PK profile of D-DOT was evaluated in rhesus monkeys [32]. In a single-dose 33.3 mg/Kg D-DOT was administered *via* intravenous and oral route. The PK studies demonstrated that D-DOT was more bioavailable than AZT and expressed an average serum half-life of 2.16 h. Also, a rapid absorption of D-DOT was observed and was almost completely absorbed by the rate constant of 2.7 h^−1^. The average renal and systemic clearance of D-DOT were 0.18- and 0.36-liter/h/kg, respectively. Within 8 h, 4–18% of the administered D-DOT (either by oral or IV route) was excreted through urine as its glucuronide conjugate, indicating an efficient systemic clearance.

The mechanism of action studies of D-DOT suggest that it converts into active DOT-TP and acts as a viral DNA chain-terminator [25]. It was also examined that D-DOT exclusively phosphorylated by human thymidine kinase-1 (TK-1) but not by other pyrimidine nucleoside kinases, including the mitochondrial thymidine kinase (TK-2) [33]. Interestingly, DOT was not a substrate for the host mitochondrial enzyme (TK-2), indicating that it lacks mitochondrial toxicity [33]. The biochemical studies suggest that compared to other approved nucleoside analogs, D-DOT exhibits greater resilience against thymidine analog-associated mutations (TAMs), as well as the M184V and K65R mutations, which are commonly observed in HIV RT and are known to compromise the efficacy of NRTI-based therapies [25]. The potent antiviral activity of D-DOT against lamivudine- and zidovudine-resistant HIV-1 mutants, and its excellent PK profile, suggest that D-DOT and related analogs may hold significant potential for preclinical/clinical development as a promising candidate for the treatment of drug-resistant HIV infections. To date, the development of D-DOT has been halted; however, further evaluation of various phosphate ester and phospholipid prodrug strategies remain warranted. These approaches may enhance its PK profile, including its activity against HIV. Furthermore, structural modification at the 5-position of D-DOT may yield new analogs with potential antiviral activity against emerging viruses.

**Figure:**
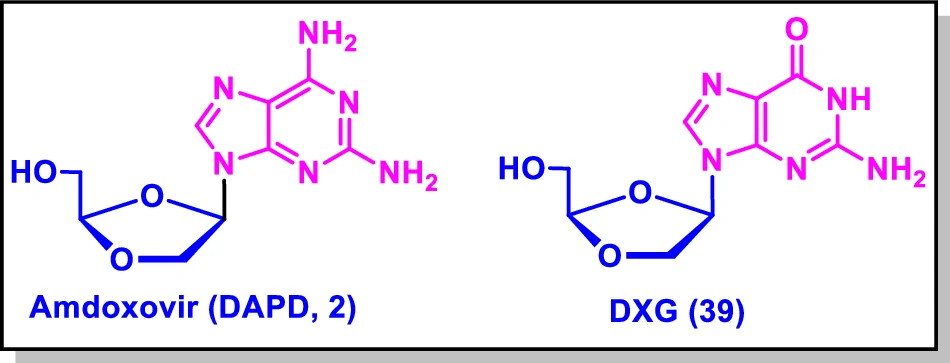

#### Amdoxovir (DAPD, 2)

The chemical name of amdoxovir is [(2 *R*,4 *R*)-4-(2,6-diaminopurin-9-yl)-1,3-dioxolan-2-yl] methanol [34]. It is also known as 1-*β*-D-2,6-diaminopurine dioxolane (DAPD, **2**). DAPD is an NRTI with potent activity against wild-type HIV-1 and it maintains its activity against key reverse transcriptase or thymidine kinase mutants [8]. It is a 6-amino prodrug, which in vivo converts to (-)-*β*-D-dioxolane guanine (DXG, **39**) [3]. Due to the limited solubility of DXG, the prodrug DAPD was synthesized to enhance its solubility [3]. Antiviral data highlight its utility as a standalone or in combination with other approved NRTIs, including enfuvirtide [35], zidovudine [36], inosine monophosphate dehydrogenase (IMP) inhibitors mycophenolic acid (MPA), and ribavirin (RBV) [37] against wild-type and drug-resistant HIV variants. While DAPD was advanced to Phase II clinical trials, it was subsequently withdrawn from further development due to safety concerns [9, 38].

**Scheme 5:**
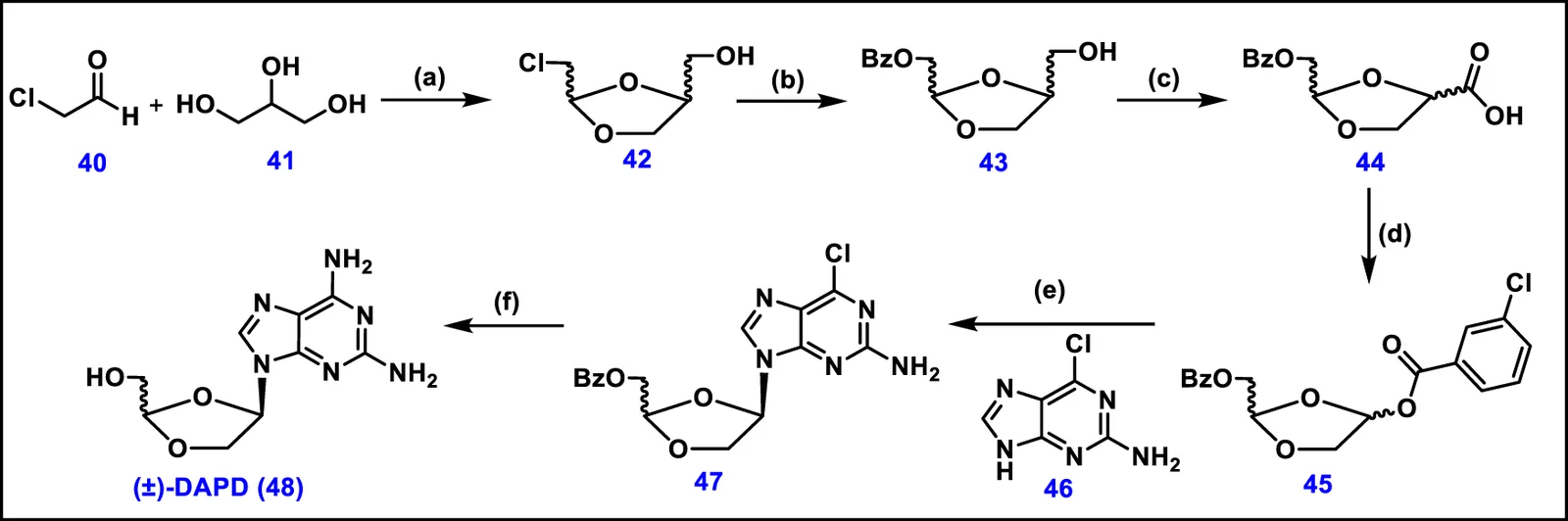
Initial synthesis of racemic (±)-DAPD **(48)**. *Reagents and conditions:* (a) Reflux 115 °C; (b) Potassium benzoate, 18-crown-6, DMF; (c) Pyridinium dichromate, DMF; (d) Ethyl chloroformate, triethylamine, *m-*CPBA, DCM; (e)TMS-Cl, hexamethyldisilazane (HMDS), TMSOTf, dichloroethane (DCE); (f) NH_3_, CH_3_OH

Belleau et al [39] has reported the first synthetic protocol for racemic (±)-DAPD. The synthesis began with the condensation of 2-chloroacetaldehyde **40** with glycerol **41** to afford the racemic 2-chloromethyl-4-hydroxymethyldioxolane intermediate **42** (Scheme 5). Compound **42** was treated with potassium benzoate in the presence of the phase-transfer catalyst 18-crown-6 ether in DMF to afford benzoate ester **43**. Hydroxy of **43** was oxidized with pyridinium dichromate in DMF to give carboxylic acid intermediate **44**.

Compound **44** was treated with ethyl chloroformate, followed by Baeyer-Villiger oxidation with meta-chloroperoxybenzoic acid (*m-*CPBA) to furnish key intermediate **45**. 6-chloro-2-aminopurine **46** was condensed with **45** in the presence of hexamethyldisilazane (HMDS), trimethylsilyl chloride (TMS-Cl), and TMSOTf to produce coupled nucleoside benzyloxy-protected 6-chloro-2-amino purine dioxolane **47**. Compound **47** was treated with a methanolic ammonia solution under pressure at 110 °C to afford the final racemic (±)-DAPD (**48**).

**Scheme 6:**
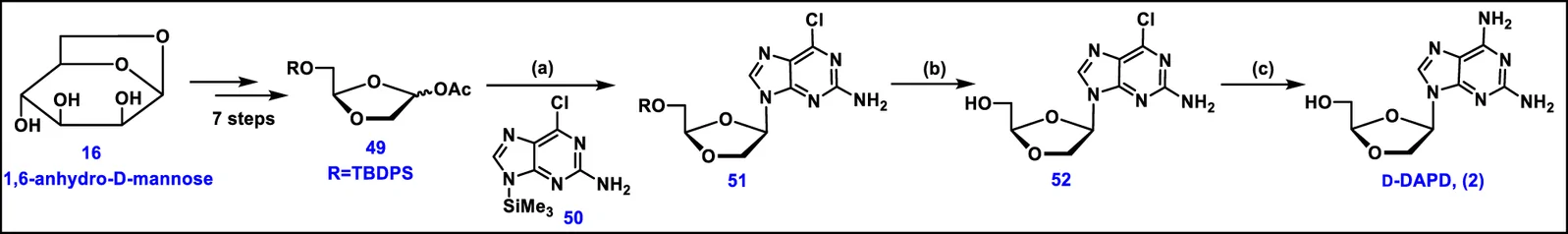
Synthesis of chiral pure D-DAPD *via* intermediate **49**. *Reagents and conditions:* TMSOTf, DCM; (b) *n*-Bu_4_NF, THF; (c) NH_3_, EtOH

The chiral pure synthesis of D-DAPD was achieved by Chu et al, starting from 1,6-anhydro-D-mannose and involving 7 steps to construct the chiral pure D-dioxolane acetate intermediate (**49**) [26]. Acetate **49** was coupled with silylated purine **50** in the presence of TMSOTf in DCM to produce nucleoside **51**. TBDPS of **51** was deprotected by treatment with tetrabutylammonium fluoride solution (TBAF or *n*-Bu_4_NF) in THF to afford compound **52**, which, upon treatment with methanolic ammonia under pressure, afforded the final chiral pure D-DAPD (Scheme 6) [34]. This reported synthesis was lengthy and involved several protections, deprotection, and oxidation steps, and was not suitable for the scalable synthesis of chiral D-DAPD.

**Scheme 7:**
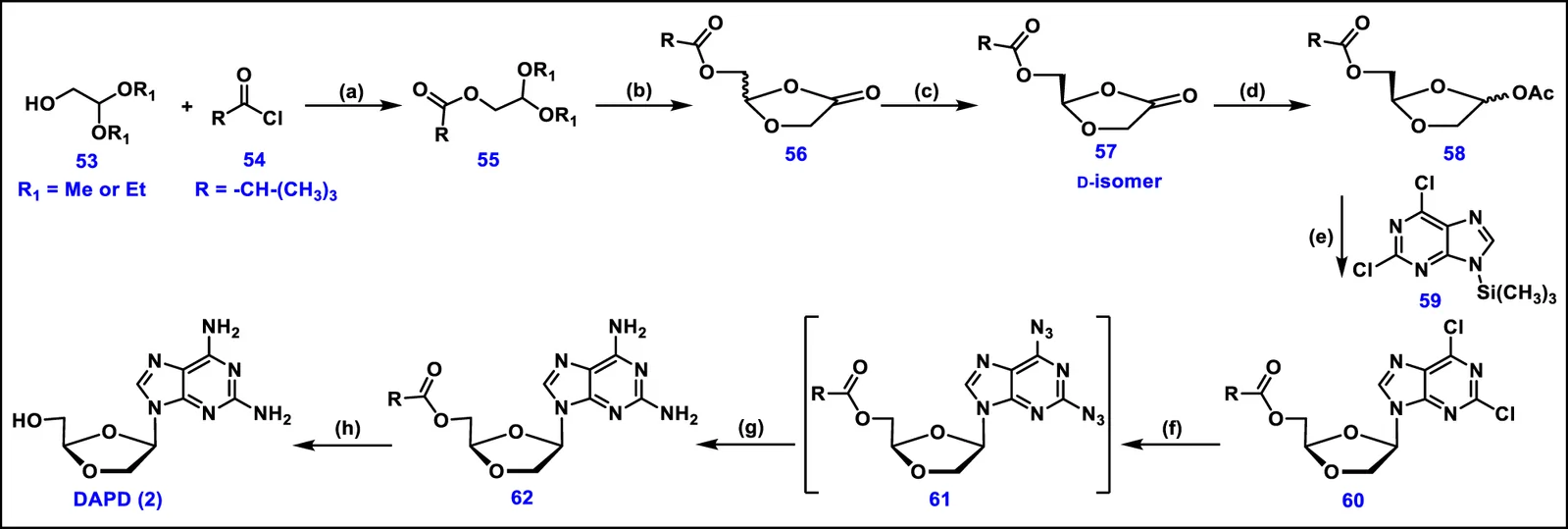
Optimized commercial synthetic process for chiral D-DAPD (**2**). *Reagents and conditions* (a) DMAP, Et_3_N, *tert*-butylmethyl ether; (b) *α*-hydroxy acetic acid, BF_3_^.^Et_2_O, acetonitrile; (c) D- and L-racemic chiral separation *via* chiral chromatography; (d) LiAlH(OBu^t^)_3_, (Ac)_2_O, THF; (e) TMSOTf, DCM; (f) Sodium azide (NaN_3_), DMF; (g) H_2_, Pd/C, DMF; (h) 2 N ammonia (NH_3_) solution or propyl amine in methanol

Furthermore, efforts were made by Belleau [40], Liotta, and Schinazi et al [41] to develop synthesis of chiral pure DAPD; however, the use of toxic reagents and oxidative steps has restricted all invented processes for the commercial development of DAPD. Later, Sznaidman et al reported an improved synthesis of chirally pure D-DAPD, which was viable for large-scale synthesis of DAPD (Scheme 7) [10]. The developed process was devoid of oxidative steps, which was suitable for the commercial development of DAPD. The racemic synthesis of ketone **56** was initiated by esterification of 2,2-dialkoxy ethanol **53** with isobutyryl chloride **54** to construct **55**. Appropriate 2,2-dialkoxyethanol **53** was treated with the corresponding acid chloride **54** in the presence of 4-dimethylaminopyridine (DMAP) and triethylamine (Et_3_N), in *tert*-butylmethyl ether to produce ester **55**. Compound **55** was condensed with *α*-hydroxy acetic acid in the presence of boron trifluoride diethyl etherate (BF_3_^.^Et_2_O) in acetonitrile to give a cyclized racemic mixture of (±)-D- and L- dioxolane ketone **56**.

Further, *via* chiral chromatographic separation, both pure chiral D- and L- isomers of **56** were separated. Desired D-isomer **57** was treated with lithium tri-*tert*-butoxyaluminum hydride [LiAlH(OBu^t^)_3_ (LTBA)] followed by acetic anhydride to produce a diastereomeric mixture of D-dioxolane acetate intermediate **58**. Coupling of **58** with 2,6-dichloropurine under Vorbrüggen conditions furnished a mixture of *β*-desired and *α*-undesired nucleosides. Compound **58** was treated with silylated purine **59** in the presence of TMSOTf in DCM to afford *β*/*α* isomeric mixture of compound **60**. Desired dichloro isomer of **60** was converted to diamino compound **62** in two steps. First, **60** was treated with sodium azide (NaN_3_) in DMF to replace the chloro with azide, affording the 2,6-diazido intermediate **61**, which, upon hydrogenation on Pd/C under a hydrogen atmosphere, afforded the 2,6-diamino compound **62**. Eventually, **62** was treated with either methanolic ammonia solution or propylamine to produce the desired compound, DAPD (**2**), in good yield.

### Antiviral activity of DAPD (2) and DXG (39)

Preliminary in vitro antiviral evaluation of DXG and its 6-amino prodrug DAPD showed potent anti-HIV activity against both wild-type and HIV RT mutants [3, 42]. DAPD was also found to be effective against wild-type and drug-resistant hepatitis B virus (HBV) variants [42, 43]. In initial screening, DAPD showed an EC_50_ of 0.7 µM in PBM cells (PBMC), with no cytotoxicity up to CC_50_ > 100 µM [34]. After this exciting finding, the antiviral potency of DAPD and DXG was thoroughly studied in various cell lines, and DAPD exhibited 12.5, 4.0, and 0.97 µM activity in MT2, PBMC, and CBMC cells, respectively (Table 2). DXG demonstrated greater antiviral potency than DAPD in MT2, PBMC, and CBMC cells.

**Table 2:** Antiviral activity of DAPD

Compounds	HIV-1 Strain LAI (LAV or IIIB)				
	HIV-1 Strain	EC_50_ (μM)	Cells	CC_50_ (µM)	References
DAPD	LAI	0.7	PBMC	>100	[34]
	LAI	12.5 ± 2.5	MT2	>1000	[3]
	LAI	4.0 ± 2.2	PBMC	>1000	[3]
	LAI	0.97 ± 0.092	CBMC	>100	[42]
	LAI	0.54 ± 0.29	MT-2	ND	[42]
	LAI	0.94	MT-4	ND	[42]
	LAI	1.37	Jurkat	ND	[42]
	LAI	0.075	H9	ND	[42]
DXG	LAI	3.4 ± 0.9	MT2	>1000	[3]
	LAI	0.25 ± 0.17	PBMC	>1000	[3]
	LAI	0.046 ± 0.017	CBMC	>100	[42]
	LAI	0.085 ± 0.026	MT-2	ND	[42]
	LAI	0.051	MT-4	ND	[42]
	LAI	0.34	Jurkat	ND	[42]
	LAI	0.06	H9	ND	[42]
	HXB2	0.5 ± 0.3	MT2	ND	[44]
	NL4-3	1.6 ± 1.1	MT2	ND	[44]

EC_50_: effective concentration of drug required to reduce viral growth by 50%

CC_50_: concentration of drug required to reduce growth of cell by 50%

ND: not determined

In extensive in vitro cytotoxicity studies, DAPD and DXG exhibited no detectable toxicity in human T-cell lines at concentrations exceeding 100–1000 µM (CC_50_), and also no mitochondrial toxicity was detected [3]. In contrast, the FDA-approved antiretroviral drug AZT demonstrated measurable toxicity in the same cell lines [3]. The data suggest that DXG is equipotent to FDA-approved lamivudine and approximately 5–10-fold less potent than AZT. Notably, further antiviral evaluations demonstrated that both DAPD and DXG retained antiviral activity against multiple 3TC- and AZT-resistant mutants and maintained efficacy against a panel of recombinant HIV-1 strains harboring resistance to 3TC and AZT [42]. Interestingly, recombinant viruses and clinical HIV-1 isolates from patients treated with NRTIs and/or non-nucleoside reverse transcriptase inhibitors (NNRTIs) remained susceptible to inhibition by DXG [44]. Against the HXB-2 and NL4-3 viral isolates derived from clinically treated patients, DXG demonstrated in vitro EC_50_ of 0.5 and 1.6 µM, respectively, in MT-2 cells (Table 2). In resistance studies, novel S68Δ deletion in HIV RT displayed 10-30 fold resistance to DAPD [45] and HIV RT with K65R [46], L74V, and/or Q151M were also found less susceptible to both DAPD and DXG [47]. Mechanistically, DAPD is deaminated by adenosine deaminase to yield the guanosine dioxolane analogue (DXG) [3]. However, the rate of DAPD deamination is markedly slower than that of the endogenous adenosine nucleoside. After deamination, DXG undergoes phosphorylation steps to form DXG-monophosphate (DXG-MP), DXG-diphosphate (DXG-DP), and DXG-triphosphate (DXG-TP). DXG-TP competes with the natural substrate 2ʹ-deoxyguanosine triphosphate (dGTP) for binding to HIV RT and effectively incorporates into elongating viral DNA, thereby causing chain termination [42]. The PK studies of DAPD and DXG were performed in the woodchuck (*Marmota monax*) animal model. The DAPD was administered at 20 mg/kg intravenously (IV) and orally, and the concentrations of nucleosides DAPD and DXG in plasma and urine were determined by HPLC. In intravenous administration, the half-life (T_1/2_) time of DAPD was found to be 6.7 h in comparison to DXG (T_1/2_ = 17.5 h), which suggests DXG has greater retention in the body than DAPD [48]. The mean total clearance (CL_T_) of DAPD was 0.33 ± 0.14 liter h/kg, and the steady state volume of distribution (V_ss_) of DAPD was found to be 1.76 ± 0.65 L/kg. The oral bioavailability of DAPD was measured in a range of 3.7 to 8.2%; however, the availability of DXG following the oral administration of DAPD was 10.5–53% [48].

Considering its favorable safety profile and potent antiviral activity against multiple HIV RT mutants, including drug-resistant variants, DAPD was selected for further preclinical and clinical development. In addition, DAPD was evaluated in combination therapies to develop effective treatments for HIV. In vitro, DAPD in combination with enfuvirtide (T-20) demonstrated synergistic antiviral activity against both sensitive and resistant clinical isolates, with significant additive effects and improved EC_50_ values [35]. Notably, in vitro, a combination of MPA and RBV, inosine monophosphate dehydrogenase inhibitors, reduces the EC_50_ of DXG by 10-fold. Also, the combination of MPA, RBV, and DAPD was effective against a mutant virus (K65R/Q151M) fully resistant to inhibition by DAPD [37]. Furthermore, in clinical trials, the combination of AZT and DAPD demonstrated synergistic efficacy, resulting in greater reductions in viral load than DAPD monotherapy, while enabling a lower effective AZT dose when combined (*clinical trial: NCT00432016*) [36]. In clinical trial, DAPD was evaluated at doses of 200, 300, and 500 mg twice daily. Data revealed that, in week 12, HIV-1 RNA levels decreased by a mean of 1.5 log10 copies/mL in the 300 mg group, and by 0.75 log10 copies/mL in the 500 mg group. However, five subjects demonstrated lens opacities during the study [49]. Additionally, preliminary toxicity assessments of DAPD revealed obstructive nephropathy resulting from drug crystallization within the renal tubules in some animals, and lens opacities were observed in both animal and human studies. Consequently, DAPD was discontinued from further clinical development due to these toxicities (clinical trial identifiers *NCT01737359* and *NCT01738555*) [49]. Subsequently, several 6-amino-modified prodrug strategies targeting DAPD were explored; however, none progressed to clinical phase [8].

**Figure:**
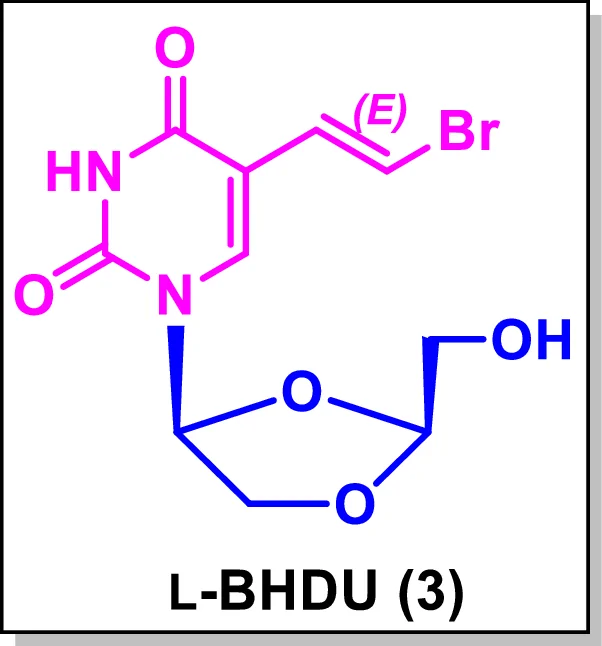

#### L-BHDU (3)

5-((*E*)-2-bromovinyl)-1-((2*S*,4*S*)-2-(hydroxymethyl)-1,3-dioxolan-4-yl)pyrimidine-2,4(1*H*,3*H*)-dione, also known as L-BHDU, is an unnatural L-dioxolane nucleoside that exhibits potent antiviral activity against VZV and herpes simplex virus (HSV) [15, 50]. The VZV causes chickenpox on primary infection and zoster (shingles) on reactivation from latency [51]. The Centers for Disease Control and Prevention (CDC) reported that approximately 1 million cases of zoster occur annually in the United States (U.S.) [52]. People over the age of 50, organ transplant patients, and individuals living with immunocompromised conditions or infection with HIV are at higher risk of VZV infection and its reactivation. Acyclovir (ACV), valacyclovir (VACV), and famciclovir (FCV) are FDA-approved drugs for the treatment of VZV infections [53]. Another drug, brivudine (BVDU), has been approved in Europe to treat VZV infections and expressed strong antiherpetic activity [54]. Despite its potency against VZV and HSV, BVDU has raised safety concerns. In the liver, BVDU is metabolized to bromovinyluracil (BVU), which inhibits the action of the dihydropyrimidine dehydrogenase (DPD) enzyme. DPD is required for the conversion of thymidine to uracil, and drug 5-fluorouracil (5-FU) is also catabolized *via* DPD [55]. Therefore, the use of BVDU reveals harmful interaction with 5-FU in cancer-treating patients. Similar to BVDU, L-BHDU belongs to a series of *E*-5-bromovinyl analogs in which an L-dioxolane ring replaces the ribose ring [7]. L-BHDU demonstrated potent antiherpetic activity against VZV [7, 15]. L-BHDU maintains both in vitro and in vivo antiviral potency against VZV in several cell lines including human retinal pigment epithelial cell line (ARPE-19), primary human foreskin fibroblasts (HFFs), skin organ culture (SOC) and in SCID-Hu mice with skin xenografts [15]. Additionally, L-BHDU was found to be safer and has not expressed any drug-drug interactions for the metabolism of 5-FU [15]. Currently, L-BHDU and its phosphate ester prodrug POM-L-BHDU-MP are under preclinical and clinical investigation to treat shingles, oral and genital herpes [7, 18].

**Scheme 8:**
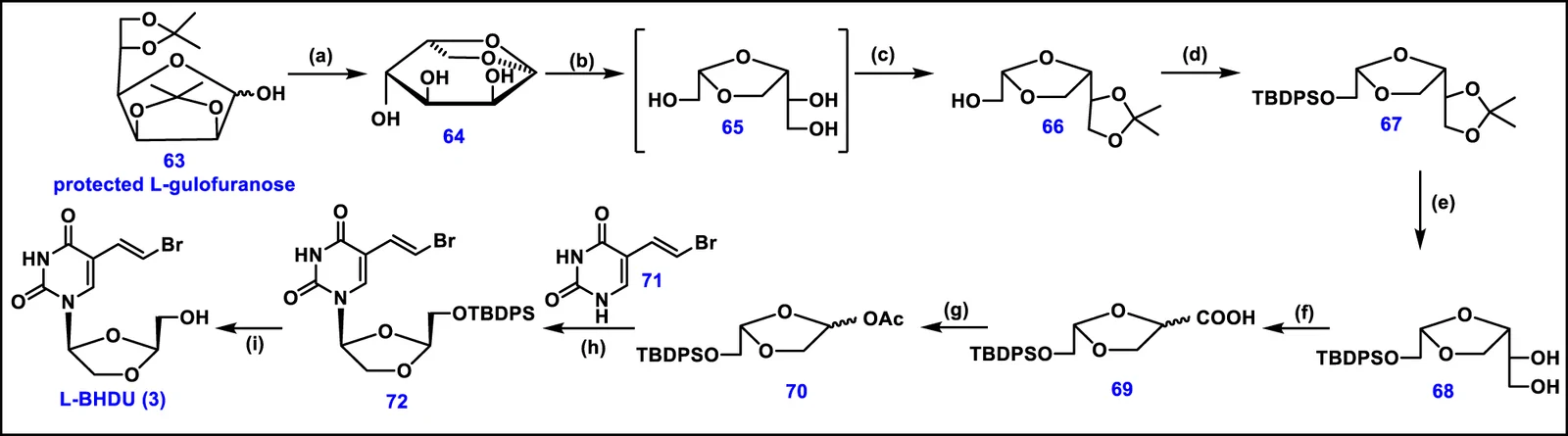
Synthesis of L-BHDU *via* isopropylidene-protected L-gulofuranose (**63**). *Reagents and conditions*: (a) 0.5 N HCl; reflux; (b) i) NaIO_4_, H_2_O, MeOH; ii) NaBH_4,_ MeOH; (c) *p-*TsOH, acetone; (d) TBDPSCl, imidazole, DMF; (e) *p*-TsOH, MeOH; (f) NaIO_4_, RuO_2_, CH_3_CN, CCl_4_, H_2_O (2:2:3); (g) Pb(OAc)_4_, pyridine; (h) TBDMSOTf, 2,4,6-collidine, DCM; (i) TBAF, CH_3_CN

The initial synthesis of L-BHDU was accomplished by Choi et al [50, 56] *via* protected-L-gulofuranose **63**. First, compound **63** was treated with 0.5 *N* HCl to afford L-l,6-anhydrogulopyranose intermediate **64**, which, upon oxidative treatment with NaIO_4_, followed by NaBH_4_ reduction, afforded dioxolane-triol intermediate **65** (Scheme 8).

The *cis* diol of dioxolane **65** was protected with isopropylidene group. **65** was treated with acetone in the presence of catalytic *p*-TsOH to furnish the isopropylidene-protected compound **66**. Furthermore, the hydroxy group of **66** was protected with *tert*-butyldiphenylsilyl (TBDPS) chloride. Intermediate **66** was treated with TBDPSCl in the presence of imidazole in DMF to give protected compound **67**. Isopropylidene deprotection of **67** was carried out with *p*-TsOH in methanol to produce **68**, which on oxidation with NaIO_4_ and RuO_2_ in a mixture of ACN-carbon tetrachloride (CCl_4_) and water (2:2:3) produces carboxylic acid **69**. Compound **69**, on treatment with Pb(OAc)_4_ in pyridine gives key acetate intermediate **70**. The crucial intermediate **70** was condensed with 5-(*E*)-bromovinyluracil **71** in the presence of TBDMSOTf, 2,4,6-collidine, and trimethylsilyl iodide (TMSI) to generate coupled nucleoside **72**. The final TBDPS deprotection of **72** was carried out using TBAF in THF, affording the desired compound L-BHDU (**3**) in good yield.

**Scheme 9:**
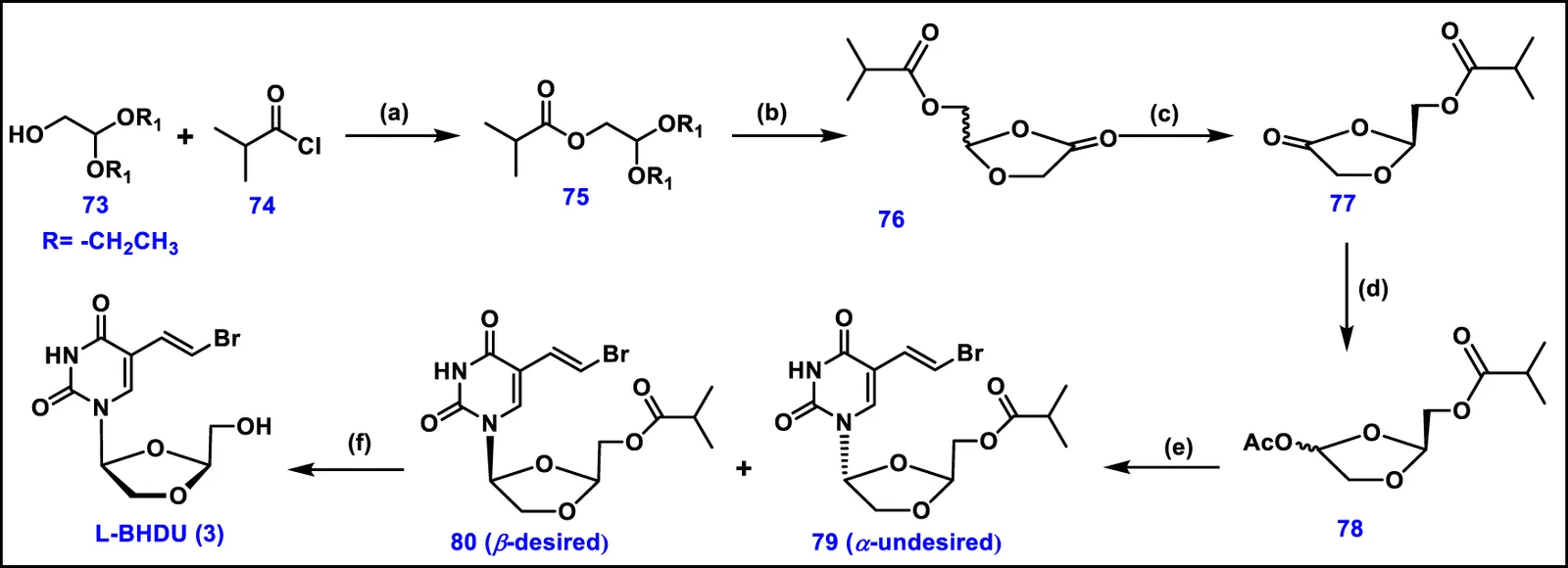
Synthesis of L-BHDU *via* the acetate intermediate **78**. *Reagents and conditions* (a) DMAP, Et_3_N, *tert*-butylmethyl ether; (b) *α*-hydroxy acetic acid BF_3_^.^Et_2_O, acetonitrile (ACN); (c) D and L racemic chiral separation *via* chiral chromatography; (d) LiAlH(O^t^Bu)_3_, (Ac)_2_O, THF; (e) TBDMSOTf, 2,4,6 collidine, TMSI, DCM; (f) 2 *N* ammonia (NH_3_) solution in methanol

Later, a revised synthesis of L-BHDU was performed, following the synthetic procedure developed for DAPD by Snzaidman et al [10]. The revised method reported in Scheme 9 avoids the use of oxidation steps involving the reagents NaIO_4_ and RuO_2_, which are tedious and challenging for the scalable synthesis of L-BHDU. The synthesis commenced with the esterification of 2,2-diethoxyethanol **73** with isobutyryl chloride **74** to construct the intermediate **75**. 2,2-diethoxyethanol **73** was treated with isobutyryl chloride **74** in the presence of DMAP and Et_3_N, in *tert*-butylmethyl ether to produce compound **75**. Compound **75** was condensed with *α*-hydroxyacetic acid to afford the cyclized racemic (±)-D- and L- dioxolane ketone **76**. The reaction of **75** with *α*-hydroxy acetic acid in the presence of BF_3_^.^Et_2_O in acetonitrile construct racemic (±) dioxolane **76**, which, upon chiral chromatographic separation, produces chiral pure L-dioxolane intermediate **77**.

The treatment of ketone **77** with LiAlH(OBu^t^)_3_ followed by in situ addition of acetic anhydride produces a diastereomeric *α*/*β* acetate dioxolane intermediate **78** in a ratio of 2:1. Furthermore, coupling of **78** with bromovinyl uracil base was carried out in the presence of TBDMSOTf, 2,4,6 collidine, and TMSI in DCM to produce compound **79** (*α*-undesired) and **80** (*β*-desired). Desired *isomer* **80** was treated with 2 N ammonia (NH_3_) solution in methanol to yield the final targeted L-BHDU (**3**).

**Scheme 10:**
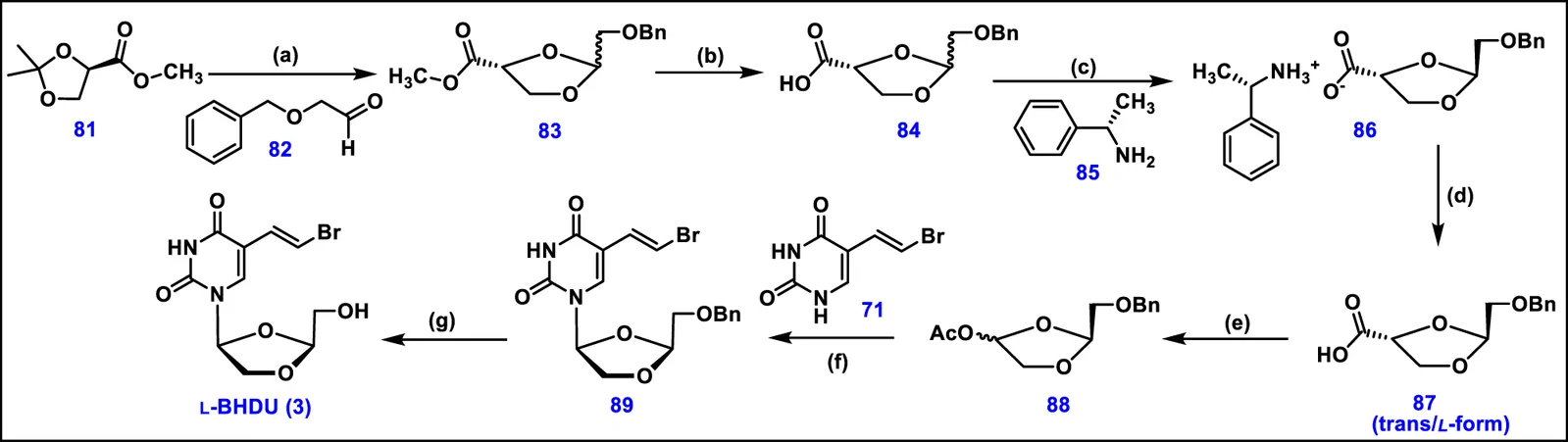
Synthesis of L-BHDU *via* methyl-*R*-dioxolane-4-carboxylate (**81**). *Reagents and conditions:* (a) Dowex 50W-X8, toluene; (b) 1 M aq. LiOH, THF; (c) i) (*S*)-phenylethylamine, acetonitrile; (ii) EtOAc:IPA (3:1); (d) 1 N aqueous HCl solution; (e) Mn(OAc)_2_, (diacetoxyiodo)benzene, acetic acid 2-Me-THF or Pb(OAc)_4_ pyridine acetonitrile; (f) TBDMSOTf, 2,4,6-collidine, TMSI, DCM; (g) BCl_3_, DCM

Furthermore, to support the preclinical and clinical development of L-BHDU, Kothapalli et al [19, 57] developed a commercial method for its large-scale synthesis. The reported synthetic method was devoid of the chiral chromatographic separation of the crucial racemic (±) D- and L-dioxolane intermediate **87** in Scheme 10, which is economical for the scalable synthesis of L-BHDU. The pure L-dioxolane was synthesized from a chiral starting material, methyl-*R*-2,2-dimethyl-1,3-dioxolane-4-carboxylate **81**. The synthesis was initiated by reacting **81** with 2-benzyloxyacetaldehyde (**82**) in the presence of Dowex 50W-X8 resin in toluene, affording dioxolane **83**. The methyl ester of **83** was hydrolyzed with 1 M aqueous lithium hydroxide (LiOH) solution in THF to give racemic (±) carboxylic acid intermediate **84**. Furthermore, the racemic mixture of **84** was resolved to pure D- and L-dioxolane *via* diastereomeric salt formation. Racemic (±) compound **84** was treated with (*S*)-phenylethylamine in acetonitrile, which gives a white salt of the acid amine as a precipitate of compound **86**. To enhance the diastereomeric excess (*de*), compound **86** was resuspended in a mixture of EtOAc-isopropanol (IPA) [3:1] to render the high *de* intermediate **86**. Acid amine salt **86** was neutralized with 1 *N* hydrochloric (HCl) solution to yield pure L-dioxolane **87** with more than 99% enantiomeric excess (*ee*). The conversion of acid **87** to acetate **88** was first carried out using Pb(OAc)_4_; however, at the bulk scale, Pb(OAc)_4_ is a toxic reagent and is not acceptable for the synthesis of clinical candidates or drug-like moieties. Considering this, the conversion of **87** to **88** was revisited, and key intermediate **88** was synthesized by the conversion of acid **87** with manganese(II) acetate [Mn(OAc)_2_] in the presence of (diacetoxyiodo)benzene and acetic acid in 2-methyl-THF [57]. Finally, coupling of **88** with bromovinyl uracil **71**, affords benzyl-protected compound **89**, which, upon treatment with boron trichloride (BCl_3_) in DCM, yields the final target compound L-BHDU **(3)**.

**Scheme 11:**
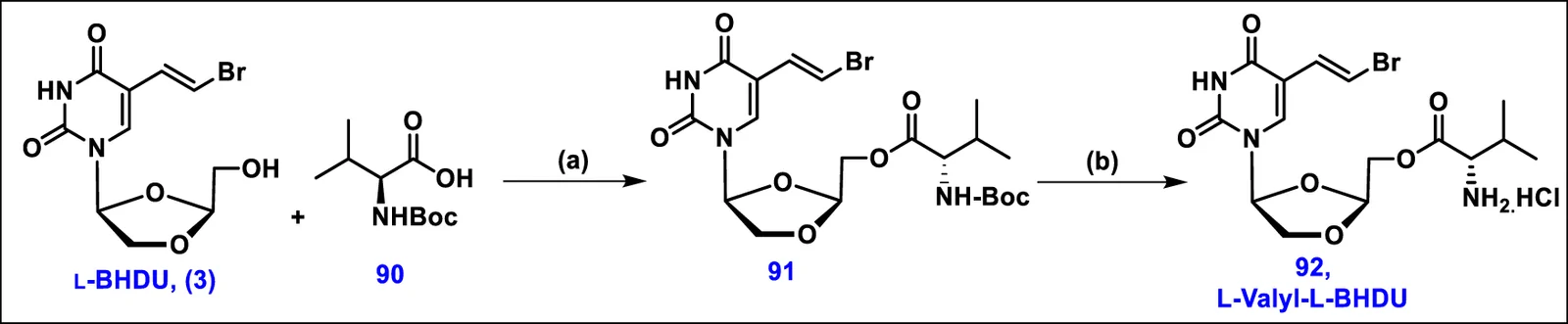
Synthesis of L-valyl-L-BHDU prodrug. *Reagents and conditions:* (a) DIC, DMAP, DCM; (b) 1 M HCl in ether, MeOH

L-BHDU is not orally active. To enhance the PK profile of L-BHDU, Singh et al [7] employed various prodrug approaches to increase its bioavailability, cellular uptake, and cell penetration. L-valyl amino ester prodrug of L-BHDU has revealed potential activity against VZV. Synthesis of L-valyl-prodrug of L-BHDU was achieved by condensation of L-BHDU with *N*-Boc-protected L-valyl amino acid in the presence of *N*,*N*′-diisopropylcarbodiimide (DIC) and catalytic DMAP in DCM to render compound Boc-protected compound **91** (Scheme 11). The Boc deprotection of **91** was carried out by 1 M HCl in methanol to furnish final L-valyl-L-BHDU **92** as an HCl salt [7, 15].

**Scheme 12:**
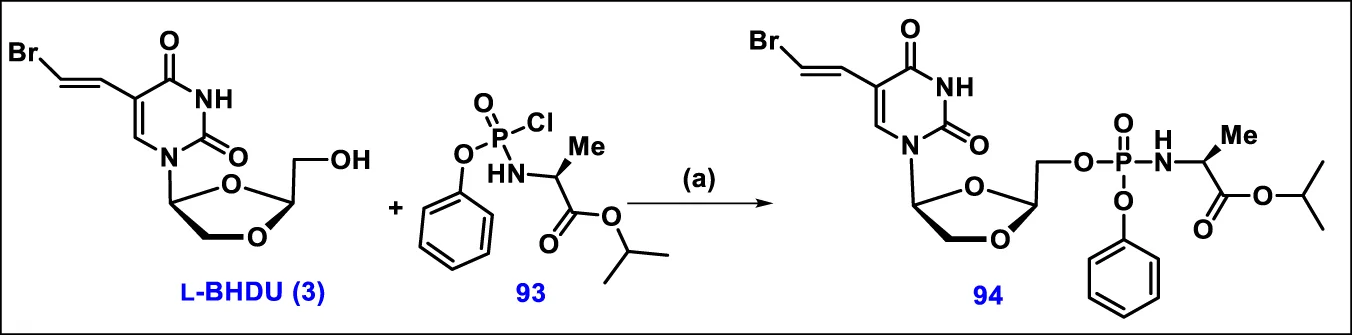
Synthesis of phosphoramidate prodrug (**94**) of L-BHDU. *Reagents and conditions:* (a) NMI, THF

Often in nucleoside drugs, the first monophosphorylation step is a roadblock to converting them into their active triphosphate form. To overcome the hurdle of first-step rate-limiting mono-phosphorylation, McGuigan et al has invented the concept of phosphoramidate prodrugs [58]. Mechanistically, aryloxy phosphoramidate prodrugs in situ in cells generate nucleoside monophosphates (NMP) by chemical and enzymatic degradation. Phosphoramidate prodrugs are also proven to have better PK properties with enhanced cellular uptake and bioavailability [59]. Considering these benefits, phosphoramidate (**94**), phosphate ester (POM-L-BHDU-MP, **96**), and long-hydrocarbon chain lipophilic prodrugs of L-BHDU were synthesized [7]. The synthesis of the phosphoramidate prodrug of L-BHDU commenced with the first, formation of phosphorochloridate intermediates **93**. Phenyl dichlorophosphate was reacted with L-alanine isopropyl ester hydrochloride in the presence of Et_3_N in DCM at -78 °C, to render intermediates **93**. Subsequently, condensation of phosphorochloridate intermediate with L-BHDU in the presence of *N*-methylimidazole (NMI) in THF affords targeted phosphoramidate prodrug **94** in good yield (Scheme 12).

**Scheme 13:**
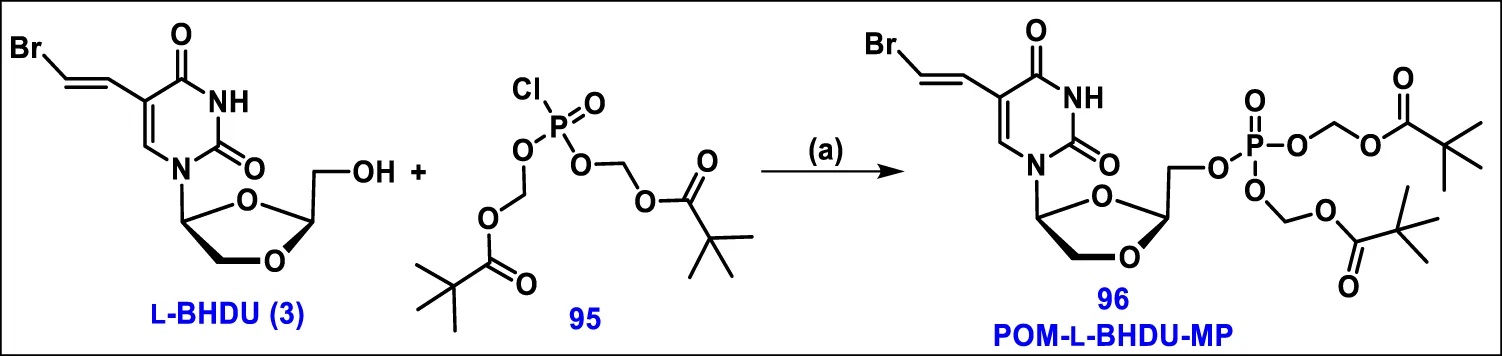
Synthesis of POM-L-BHDU-MP prodrug. *Reagents and conditions:* (a) NMI, THF

The synthesis of phosphate ester prodrug of L-BHDU was carried out by coupling of bis(POM)phosphorochloridate (**95**) [60] with L-BHDU in the presence of NMI in THF to construct POM-L-BHDU-MP (**96**, Scheme 13).

### Antiviral activity of L-BHDU and its prodrugs

Preliminary L-BHDU demonstrated promising in vitro antiviral activity against VZV. In initial screening, L-BHDU exhibited a potent antiviral profile, with an EC_50_ of 0.07 µM and a selectivity index (SI) > 1428 [50]. To confirm the antiviral potency, L-BHDU was screened against various viral strains in several cell lines and was found to be potent in all evaluations. L-BHDU showed EC_50_ values of 0.22 and 0.24 µM against VZV-BAC-Luc in HFF cells and SOC, respectively, with a high SI (Table 3).

**Table 3:** In vitro antiviral activity of L-BHDU and its prodrugs in various cell lines against different viral strains

Compounds	Viral Strains	Cell Lines	EC_50_ (µM)^a^	CC_50_ (µM)^b^	SI (CC_50_/EC_50_)^c^	References
L-BHDU (**3**)	VZV (Ellen)	HEL 299	0.07	>100	>1428	[50]
L-BHDU (**3**)	VZV-BAC-Luc	HFFs	0.22	>200	>909	[15]
L-BHDU (**3**)	VZV-BAC-Luc	SOC	0.24	ND	ND	[15]
L-BHDU (**3**)	VZV-ORF57-Luc	ARPE-19	0.026	>100	>3878	[7]
L-Valyl-L-BHDU (**92**)	VZV-BAC-Luc	HFFs	0.03	>200	>6667	[15]
L-Valyl-L-BHDU (**92**)	VZV-ORF57-Luc	ARPE-19	0.03	>100	>3322	[7]
L-BHDU-isopropyl-L-alanine-phosphoramidate (**94**)	VZV-ORF57-Luc	ARPE-19	1.69	>100	>59	[7]
POM-L-BHDU-MP (**96**)	VZV-ORF57-Luc	ARPE-19	0.036	>100	>2809	[7]

*HEL 299* human embryonic lung cells, *HFFs* human foreskin fibroblasts cells, *SOC* skin organ culture, *ARPE-19* adherent retinal pigmented epithelial cells

^a^50% inhibitory concentration 48 hpi determined by bioluminescence imaging, mean ± standard deviation from at least 3 experiments

^b^50% cytotoxic concentration at 72 h determined by neutral red assay from 3 experiments

^c^Selectivity index = CC_50_/EC_50_

In repeated screening assays using ARPE-19 cells, L-BHDU maintained its antiviral profile, with an EC_50_ of 0.26 µM and a SI greater than 3878 against the VZV-ORF57-Luc strain [7]. The L-valyl prodrug of L-BHDU (**92**) demonstrated enhanced antiviral potency, exhibiting an EC_50_ of 0.03 µM against VZV-BAC-Luc in HFF cells with no observed cytotoxicity (CC₅₀ > 200 µM). Additionally, it showed an EC_50_ of 0.03 µM against the VZV-ORF57-Luc strain in ARPE-19 cells, with a high SI [7, 15]. Notably, the L-valyl prodrug of L-BHDU retained its antiviral activity compared to the parent nucleoside. Phosphoramidate prodrug of L-BHDU (**94**), showed an EC_50_ of 1.69 µM against VZV-ORF57-Luc and was found to be less potent in comparison to L-BHDU. However, phosphate ester prodrug of L-BHDU, POM-L-BHDU-MP, expressed great antiviral activity, with an EC_50_ of 0.036 µM and a SI > 2809 [7].

Since POM-L-BHDU-MP contains a monophosphate moiety, it is designed to bypass the initial phosphorylation step that typically depends on viral thymidine kinase (TK) for unphosphorylated nucleosides [59]. Owing to this advantageous mechanism, POM-L-BHDU-MP was selected for further preclinical and clinical development.

In various altered cells and VZV strain evaluations, L-BHDU and POM-L-BHDU-MP have shown the best antiviral profiles against VZV-ORF57-Luc (Table 4). To examine L-BHDU and POM-L-BHDU-MP against viral mutants, these molecules were screened against VZV TK and TS (thymidylate synthase) mutants using standard cidofovir (CDV) and acyclovir (ACV) as positive controls [61]. These studies were performed against VZV-ORF57-Luc, including with the mutant strains, VZV-ORF57-∆TK, VZV-ORF57-∆TS, and VZV-ORF57-∆TKTS in ARPE-19 cells [61]. The EC_50_ of both L-BHDU and POM-L-BHDU-MP against cell-associated VZV were comparable or slightly higher than those obtained with cell-free VZV (Table 4). L-BHDU and POM-L-BHDU-MP were more potent against VZV-ORF57-Luc than CDV or ACV (Table 4). However, CDV demonstrated antiviral activity against VZV-ORF57-∆TK and VZV-ORF57-∆TKTS, whereas ACV did not, owing to its dependence on phosphorylation by TK. L-BHDU and POM-L-BHDU-MP exhibited EC_50_ of 0.028 µM and 0.0012 µM, respectively, against VZV-ORF57-∆TS strain, and prodrug **96** surpassed antiviral activity compared to standard CDV and ACV. Additionally, L-BHDU and POM-L-BHDU-MP were found to be not active against VZV-ORF57-∆TK or VZV-ORF57-∆TKTS [7].

**Table 4:** Antiviral activity of L-BHDU and POM-L-BHDU-MP against cell-associated wild-type and mutant VZV viruses in ARPE-19 cells [7]

	EC_50_ (µM)			
Viral Strains	Cidofovir (CDV)	Acyclovir (ACV)	L-BHDU	POM-L-BHDU-MP
VZV-ORF57-Luc	0.37	7.75	0.18	0.051
VZV-ORF57- ∆TK	0.42	>20	>2.0	>2.0
VZV-ORF57-∆TS	0.012	0.37	0.028	0.0012
VZV-ORF57-∆TKTS	1.05	>20	>2.0	>2.0

L-BHDU and its L-valyl prodrug, **92**, were evaluated in vivo using the SCID-Hu mouse model implanted with VZV-infected human fetal skin xenografts (VZV-BAC-Luc) [62, 63]. L-BHDU was administered orally at doses of 8, 15, and 150 mg/kg/day, while L-valyl-L-BHDU was given at doses of 10 and 30 mg/kg/day. Control groups included mice treated with 0.4% CMC (vehicle), acyclovir (120 mg/kg/day), or valacyclovir (120 or 200 mg/kg/day). In vivo, mice treated with L-BHDU and L-valyl-L-BHDU showed reduced viral growth; a 150 mg/kg/daily dose of L-BHDU and a 30 mg/kg/daily dose of L-valyl-L-BHDU were effective in mimicking viral growth compared with the vehicle group [15]. There is no significant in vivo toxicity observed in L-BHDU and L-valyl-L-BHDU treated mice compared to the control group. L-BHDU also expressed a dose-dependent tissue distribution in plasma, heart, spleen, liver, kidney, xenograft skin (human), and mouse skin [15]. The in vitro and in vivo data revealed that L-BHDU is more active against VZV than ACV and VACV and offer preclinical and clinical development for the treatment of herpes zoster. Additionally, catabolic studies proved that L-BHDU does not affect the metabolism of 5-FU. It was concluded that L-BHDU is an unnatural L-dioxolane analog and is not a substrate for the thymidine phosphorylases (TPase) [64] to catabolize into BVU base, which mimics the metabolism of 5-FU. Hence, the cancer-treating patients are safe with the co-treatment of 5-FU and L-BHDU [15].

The PK profiles of L-BHDU and POM-L-BHDU-MP were evaluated for stability in liver microsomes, gastric and intestinal juices. It was found that POM-L-BHDU-MP was stable in both gastric and intestinal juice [7]. However, in liver homogenate examinations, POM-L-BHDU-MP rapidly converted to L-BHDU and L-BHDU-monophosphate (L-BHDU-MP). POM-L-BHDU-MP was incubated with homogenized mouse liver and was found to be unstable and converted to L-BHDU and L-BHDU-MP [7].

To determine the oral availability of POM-L-BHDU-MP compared to L-BHDU, a single equimolar dose of POM-L-BHDU-MP or L-BHDU was administered by oral gavage to two groups of BALB/c mice. The AUC value for L-BHDU in POM-L-BHDU-MP-treated mice was approximately 2.2-fold more than that of L-BHDU-treated mice. Additionally, *C*max for L-BHDU was significantly higher after administration of prodrug POM-L-BHDU-MP than L-BHDU (Table 5) [7]. Therefore, it was concluded that POM-L-BHDU-MP exhibits better oral absorption/availability relative to L-BHDU and is a promising candidate for further drug development against VZV infection. Currently, POM-L-BHDU-MP is undergoing toxicological and extended PK analysis and may be progressed to clinical development.

**Table 5:** Pharmacokinetic (PK) profile of POM-L-BHDU-MP and L-BHDU after oral administration of 22.5 mg/kg POM-L-BHDU-MP and 11.4 mg/kg L-BHDU to mice [7]

Compounds	AUC (µg.min/mL)	*T* max (min.)	*C* max (µg/mL)
L-BHDU derived from POM-L-BHDU-MP	1680	40	10.6
L-BHDU	764	120	7.1

**Figure:**
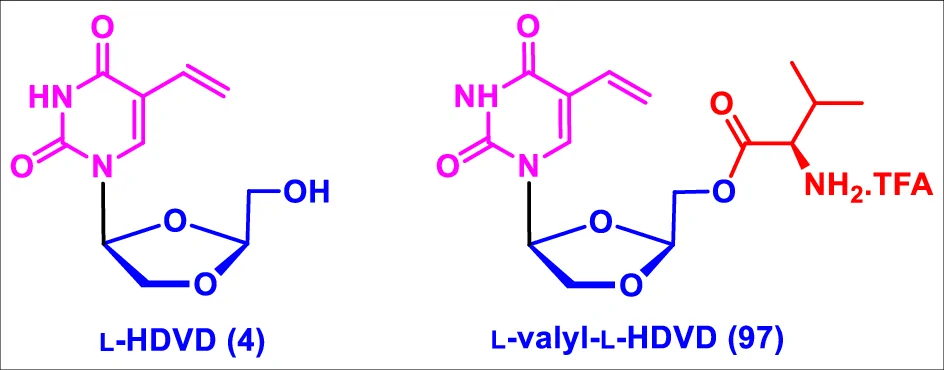

#### L-HDVD (4)

Chemically, L-HDVD, is known as L-[(2*S*,4*S*-2-(hydroxymethyl)-1,3-dioxolan-4-yl]5-vinylpyrimidine-2,4(1*H*,3*H*)-dione. It is an L-dioxolane analog that, in vitro, has shown effective and selective antiviral activity against EBV, KSHV, and HSV 1 [4]. EBV is a DNA virus that establishes life-long infection and causes mononucleosis, which is a considerable cause of morbidity in adolescents and young adults [65]. Both EBV and KSHV infections show tropism for lymphocytes and increase the risk of several cancers and autoimmune diseases [66, 67]. A recent definitive epidemiology study proves that individuals with EBV infection are 32 times more at risk for multiple sclerosis (MS) [68]. MS is a leading disease that impacts more than 2.8 million people worldwide. Notably, immunocompromised persons with cancer, HIV infected, and organ transplant patients have a higher risk of infections and reactivation of EBV and KSHV [69]. Infections of HSV 1 and 2 are a significant cause of oral and genital herpes and necessitate the discovery of new antivirals [70, 71]. In vitro, L-HDVD and its L-valyl prodrug (**97**) showed potent activity against EBV and KSHV and were more active than the FDA-approved ganciclovir and ACV [4]. It was speculated that L-HDVD selectively interacts with viral DNA polymerase, and its triphosphate (L-HDVD-TP) is anticipated to function as a competitive inhibitor with natural substrate dTTP (2ʹ-deoxy thymidine triphosphate). L-HDVD-TP may act as a DNA chain terminator by incorporating into the elongating viral DNA and blocking further extension due to its lack of the required 3ʹ-OH [4]. L-valyl-L-HDVD may demonstrate better oral availability and cellular uptake, which, after ester hydrolysis, produces L-HDVD. ACV and BVDU have demonstrated specificity to EBV TK, converting it to its active phosphate form [72]. This finding is significant, as EBV TK exhibits a narrower substrate specificity for nucleoside analogs compared to alpha (*α*)-herpesvirus TK, making it a challenging target for nucleoside-based antivirals [4]. In vitro antiviral data suggest that L-HDVD and L-valyl-L-HDVD have potential for preclinical and clinical development to treat EBV and KSHV infections and may facilitate in the cure of their associated cancers.

**Scheme 14:**
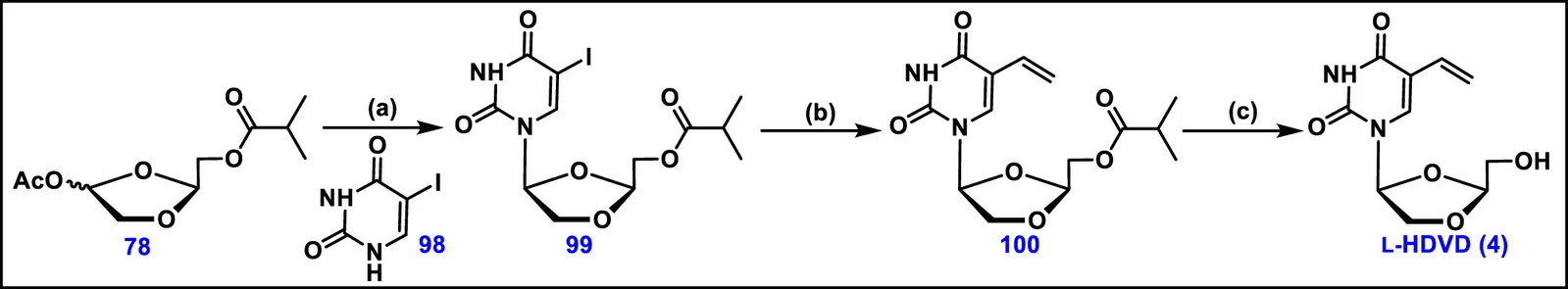
Synthesis of L-HDVD *via* chiral L-dioxolane intermediate **78**. *Reagents and conditions:*(a) TBDMSOTf, 2,4,6-collidine, TMS-I, DCM; (b) tributyl(vinyl)tin, PdCl_2_(PPh_3_)_2_, THF; (c) 7 N NH_3_ solution in MeOH

The synthesis of L-HDVD was initiated with coupling of **78** of Scheme 9 with iodouracil base **98** by using TBDMSOTf, 2,4,6-collidine, and TMSI in DCM to produce *α-* and *β*-coupled nucleosides in a ratio of 1:3. The desired *β*-nucleoside (**99**) was treated with tributyl(vinyl)tin in the presence of bis(triphenylphosphine)palladium(II)dichloride (Pd(Ph_3_P)_2_Cl_2_) in THF to furnish the vinyl nucleoside **100** [73]. Finally, 5ʹ-isobutyl acetate was deprotected by treating **100** with 7 N methanolic ammonia solution in MeOH, affording the target compound L-HDVD (**4**) in good yield (Scheme 14).

**Scheme 15:**
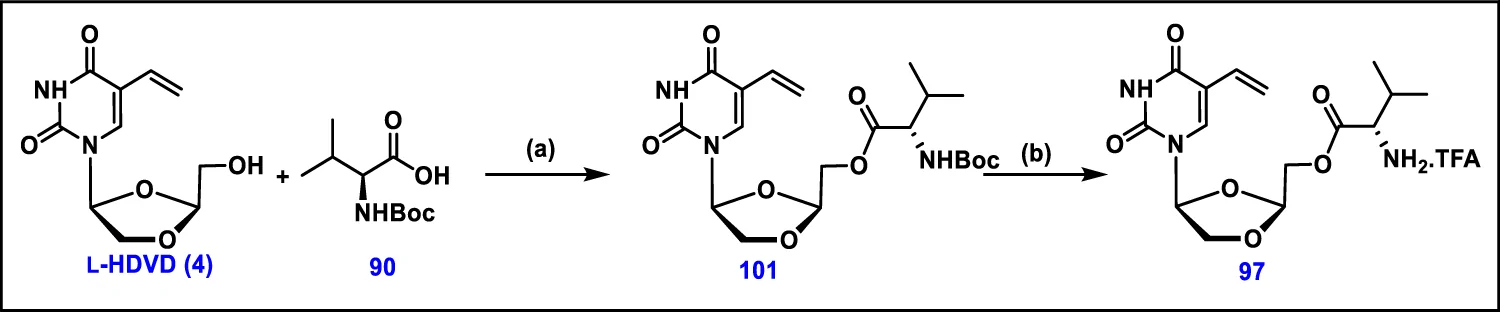
Synthesis of L-valyl-L-HDVD (**97**) amino ester prodrug of L-HDVD. *Reagents and conditions* (a) DMAP, DIC, DCM; (b) 2 M TFA solution in DCM

The synthesis of L-valyl-L-HDVD prodrug (**97**) was carried out by the condensation of L-HDVD with L-Boc-valyl amino acid (**90**, Scheme 15). L-HDVD was treated with L-Boc-valyl amino acid in the presence of DIC and DMAP in DCM to generate Boc-protected compound **101**. Compound **101** was treated with 2 M solution of TFA in DCM to furnish amino ester prodrug L-valyl-L-HDVD [4].

### Antiviral activity of L-HDVD (4)

L-HDVD was tested against various gammaherpesviruses and herpesviruses using reference FDA-approved ACV [74], BVDU [54], CDV [75, 76], and ganciclovir (GCV) [77] as controls. By using qPCR antiviral assay, L-HDVD and its valyl prodrug (**97**) were evaluated against EBV and KSHV in P3HR-1 and BCBL-1 cell lines, respectively [4]. L-HDVD demonstrated a potent activity with an EC_50_ of 0.01 µM, and L-valyl-L-HDVD expressed an EC_50_ of 0.02 µM against EBV (Table 6).

**Table 6:** Antiviral activity of L-HDVD and L-valyl-L-HDVD against EBV and KSHV

Compounds	EBV (P3HR-1 cell) inhibition				KSHV (BCBL-1 cell) inhibition			
	EC_50_ (μM)^a^	EC_90_ (μM)^b^	CC_50_ (μM)^c^	SI	EC_50_ (μM)	EC_90_ (μM)	CC_50_ (μM)	SI
L-HDVD	0.01 ± 0.003	0.07 ± 0.02	77	7700	0.09 ± 0.04	0.2 ± 0.03	100	1111
L-valyl-L-HDVD	0.02 ± 0.02	≥ 0.4	>200	>10,000	0.1 ± 0.06	0.7	>200	>2000
ACV	3.6 ± 0.4	16 ± 8	>200	>56	138 ± 67	>200	>200	>1.5
BVDU	6.9 ± 5.1	≥ 33	>200	>29	0.9 ± 0.9	2.7 ± 2.4	102 ± 42	113
CDV	2.5 ± 1.3	25 ± 31	>200	>80	1.0 ± 0.3	2.2 ± 1.3	>200	>200
GCV	4.3 ± 3.6	18 ± 2	>200	>47	6.4 ± 2.6	40 ± 30	≥200	≥32

^a and b^EC_50_ and EC_90_: effective concentration of drug required to reduce viral synthesis by 50% and 90% respectively

^c^CC_50_ : cytostatic concentration of drug required to reduce growth on uninduced cell by 50%

^d^SI : selectivity index ratio of CC_50_/EC_50_

L-HDVD was found to be moderately toxic with a CC_50_ of 77 µM; however, prodrug **97** had not expressed any toxicity up to CC_50_ > 200 µM. Both L-HDVD and the L-valyl-L-HDVD demonstrated several-fold greater antiviral potency than standard drugs, including ACV, BVDU, CDV, and GCV (Table 6). L-HDVD exhibited a SI of approximately 7700, while prodrug **97** showed an even higher SI > 10,000. On the other hand, against KSHV, L-HDVD showed an EC_50_ of 0.09 µM and a SI of 1111. In comparison, L-valyl-L-HDVD exhibited an EC_50_ of 0.1 µM without cytotoxicity (CC_50_) up to > 200 µM against KSHV with an enhanced SI of >2000. Notably, in vitro also against KSHV, both L-HDVD and its prodrug L-valyl-L-HDVD were found to be more potent than the FDA-approved drugs (Table 6).

L-HDVD was also evaluated against seven strains of human herpesviruses, including HSV-1 and -2, VZV, and human cytomegalovirus (HCMV, Table 7). The antiviral activity of L-HDVD was determined by the inhibition of virus-induced cytopathic effect (CPE) or VZV plaque formation. L-HDVD revealed excellent activity against HSV-1 KOS (EC_50_ of 0.1 μM); however, it expressed slight activity against HSV 2 with an EC_50_ of 12 μM and was inactive against acyclovir-resistant strain HSV-1 KOS/ACV^r^. It demonstrated weak activity (EC_50_ of 37 µM) against the Oka strain of VZV and was not active against HCMV without cellular toxicity, with CC_50_ > 100 µM [4].

**Table 7:** Antiviral activity of L-HDVD against various strains of HSV-1, VZV and HCMV

Compounds	Antiviral activity by virus strain EC_50_ (µM)^a^								
	HSV-1			VZV		HCMV		Cytotoxicity (μM)	
	KOS	KOS/ACV^r^	HSV 2 G	Oka (TK + )	07-1 (TK-)	AD169	Davis	CC_50_^b^	MCC^c^
L-HDVD	0.1 ± 0.06	>100	12 ± 11	37	>100	>100	>100	>100	>100
ACV	0.4 ± 0.05	170 ± 41	0.5 ± 0.3	1.5	96	ND	ND	640	>200
BVDU	0.06 ± 0	≥176	302 ± 0	0.003	60	ND	ND	471	≥150
CDV	1.2 ± 0.6	0.7 ± 0.9	1.0 ± 0.7	ND	ND	0.6	1.3	97	>150
GCV	ND^d^	ND	ND	ND	ND	11	13	196	>150

^a^EC_50_: effective concentration of drug required to reduce virus plaque formation or CPE by 50%

^b^CC_50_: cytostatic concentration of drug required to reduce growth on uninduced cell by 50% by 3 days of incubation

^c^MCC: concentration of drug required to cause a microscopically detectable alteration of normal cell morphology

^d^ND: not determined

Furthermore, L-HDVD and its prodrug **97** were evaluated against the animal gammaherpesvirus, murine herpesvirus 68 (MHV-68, Table 8) [4]. L-valyl-L-HDVD demonstrated an EC_50_ of 0.08 µM, which is slightly more potent than the parent nucleoside L-HDVD (EC_50_ of 0.1 µM), and both compounds showed high SI. L-HDVD and L-valyl-L-HDVD exhibited comparable activity to BVDU; however, both displayed substantially higher SI values than BVDU.

**Table 8:** Antiviral activity of L-HDVD and L-valyl-L-HDVD, against animal gammaherpesviruses MHV-68

Compounds	MHV-68 inhibition (NIH 3T3 cell)		
	EC_50_ (μM)	CC_50_ (μM)	SI
HDVD	0.1 ± 0.03	>200	>2000
L-valyl-L-HDVD	0.08 ± 0.04	>200	>2500
ACV	8.9 ± 8.9	71 ± 31	8
BVDU	0.1 ± 0.06	12 ± 3	120
CDV	1.3 ± 0.3	105 ± 22	81
GCV	18 ± 3.6	138 ± 39	8

It is important to note that L-HDVD and its L-valyl prodrug demonstrated comparable antiviral efficacy across all tested viruses. Both compounds also exhibited low, to no cytotoxicity in all tested cell lines and displayed high SI values. Viral TK activates L-HDVD that subsequently targets viral DNA polymerase [4]. Nucleoside analogs may also be activated by mitochondrial TK-2, which can result in host mitochondrial toxicity [78]. This is an issue associated with nucleoside-based drugs because the inhibition of dThd phosphorylation by mitochondrial TK-2 catalysis can lead to mitochondrial toxicity [79]. Therefore, L-HDVD was compared with BVDU for the inhibition of dThd phosphorylation. During the study, L-HDVD showed an approximately 500-fold decrease in its ability to inhibit TK-2–mediated dThd phosphorylation [4]. It indicates that L-HDVD has a substantially lower affinity for TK-2 and is unlikely to contribute to host mitochondrial toxicity. Additionally, L-HDVD showed stronger substrate activity with HSV-1 TK than with VZV TK, which explains why it was more active against HSV-1 than VZV [4]. After establishing the affinity of L-HDVD for viral TK, additional mutation studies were conducted using MHV-68 TK at higher L-HDVD concentrations. The studies revealed three distinct mutations in the TK gene of MHV-68 isolates that emerged following selection for L-HDVD resistance. Notably, no L-HDVD associated mutations or resistance were observed in the DNA polymerase or in the protein kinases of gamma herpesviruses [4].

Furthermore, L-HDVD in vivo evaluation was performed in BALB/c mice infected with the MHV-68 using BVDU as the standard drug [4]. Notably, MHV-68 is closely related to murine gammaherpesvirus and encodes a TK with significant homology to TKs of both EBV and KSHV [80, 81]. L-HDVD was orally administered at 200 mg/kg body weight once daily for five consecutive days, beginning on the day of infection. Alternatively, L-valyl-L-HDVD was administered at 100 mg/kg twice daily for five consecutive days. After a 6- and 12-day treatment of L-HDVD and L-valyl-L-HDVD, viral DNA loads in lung, spleen, and mediastinal lymph nodes (MLNs) were determined by qPCR. The data revealed that viral DNA loads were efficiently reduced after 6- and 12-day treatment of both L-HDVD and L-valyl-L-HDVD. Interestingly, in MLNs, neither L-HDVD nor BVDU affected viral load compared with untreated mice. No in vivo toxicities were observed for either L-HDVD or its prodrug. L-HDVD demonstrated robust inhibition of MHV-68 replication and effectively delayed the establishment of viral latency in the spleen [4]. These data indicate that L-HDVD, or its prodrug, offers substantial potential for the therapeutic management of EBV- and KSHV-associated diseases and for preventing or delaying the onset of virus-associated cancers, particularly in immunocompromised individuals.

**Figure:**
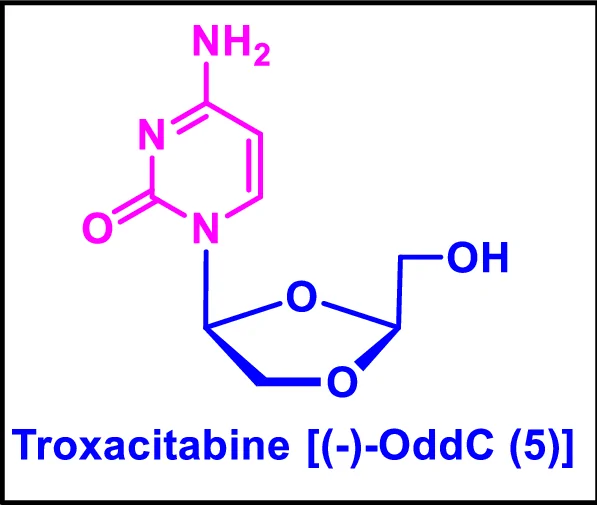

#### Troxacitabine (5)

Troxacitabine (**BCH 4556**, or **SPD 758**), originally known as L-(-)-dioxolane-cytidine or [(-)-OddC] [5]. It is the first L-dioxolane nucleoside analog that showed promising anticancer activity [23]. Originally, L-enantiomers of dioxolane- or oxathiolane-derived nucleosides were believed to be inactive drugs because they were not recognized by cellular enzymes [2]. However, the discovery of lamivudine [82], an oxathiolane-derived molecule, proved that L-dioxolane derived nucleoside analogs may have therapeutic potential [83]. Later, troxacitabine was invented as a potent anticancer agent [5, 84]. Initially, in preclinical studies, troxacitabine expressed broad-spectrum anticancer activity against solid tumors, multidrug-resistant (MDR) tumors [85], hematological malignancies [86], and chemoresistant tumors [5, 87]. Mechanistically, it undergoes sequential conversion into mono-, di-, and triphosphate forms and is subsequently incorporated into the elongating DNA chain, leading to DNA chain termination [5, 88]. Additionally, troxacitabine is not the substrate for cytidine deaminase (CDA or dCD), which is beneficial compared to other deoxycytidine nucleoside analogs [5]. It was progressed to phase II clinical trial to treat refractory lymphoproliferative neoplasms or multiple myeloma [89] as well as advanced cancer of the pancreas and gastrointestinal tumors [90]. Results from a phase II clinical study indicated that troxacitabine monotherapy provided limited clinical benefit in patients with advanced lymphoproliferative disorders or multiple myeloma. However, modification of the troxacitabine dosing regimen, particularly in combination therapy, may enhance its clinical utility in the treatment of hematologic malignancies. Currently, the phosphoramidate prodrug of troxacitabine is under investigation for the treatment of liver cancer [91].

**Scheme 16:**
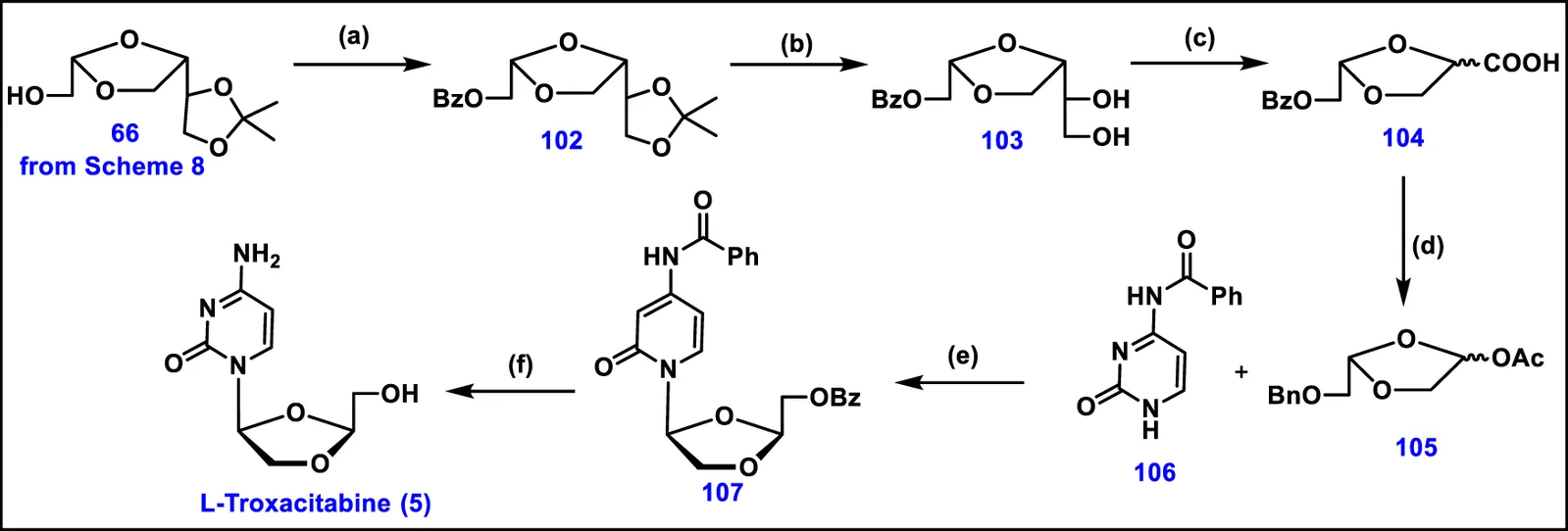
Initial synthesis of troxacitabine from intermediate **66**. *Reagents and conditions*: (a) BzCl, pyridine, DCM; (b) *p*-TsOH, MeOH; (c) NaIO_4_, RuO_2_, CH_3_CN-CCl_4_-H_2_O (2:2:3); (d) Pb(OAc)_4_, pyridine; (e) hexamethyldisilane, cat. ammonium sulfate (NH_4_)_2_SO_4_, TMSOTf, DCM, rt, 3 h; (f) NH_3_/MeOH

Initially, troxacitabine was synthesized from protected-L-gulofuranose. Protected-L-gulofuranose was converted to intermediate **66** (synthesis described in Scheme 8) [56]. Compound **66** was converted to crucial acetate intermediate **105** *via* a sequence of protection, deprotection, oxidation, and acetylation (Scheme 16**)** [92]. Compound **105** was coupled with the silylated *N*-acetylated cytosine **106** *via* HMDS in the presence of TMSOTf in DCM to produce the desired *β*-coupled compound **107**. Finally, treatment of **107** with a methanolic ammonia solution affords the final troxacitabine (**5**).

**Scheme 17:**
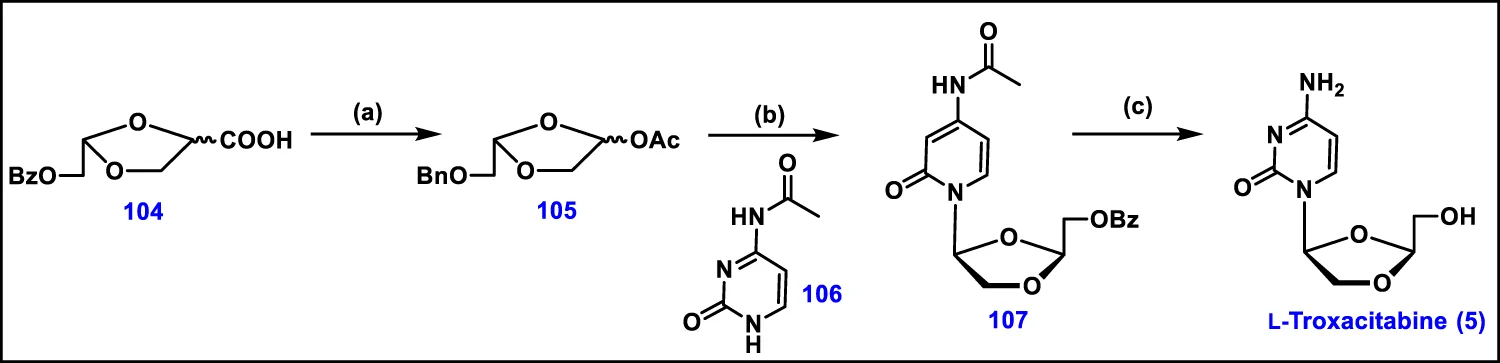
Improved synthesis of troxacitabine *via* intermediate **104**. *Reagents and conditions*: (a) (Diacetoxyiodo)benzene (DIB), I_2_, DCM; (b) TMSI, HMDS, DCM; (c) Sodium methoxide (NaOMe), methanol

To support the scalable synthesis of troxacitabine, Gregory et al [93] developed a revised synthesis by using acid intermediate **104**. Acid **104** was treated with DIB and iodine (I_2_) in DCM to produce diastereomeric acetoxy intermediate **105**, which, on coupling with *N*-acetyl cytosine **106**, in the presence of TMSI and HMDS, gives the mixture of desired (*β*)-**107** and undesired (*α*)-**107** coupled products. Final deprotection of the acetyl and benzoyl moieties were achieved by treating **107** with a solution of sodium methoxide in methanol, affording troxacitabine in good yield (Scheme 17).

### Anticancer activity of troxacitabine (5)

In preliminary evaluations, troxacitabine was first tested in multiple cell lines, including nasopharyngeal carcinoma (KB), prostate carcinoma (DU-145 and PC-3), hepatocellular carcinoma (HepG2 and 2.2.15), and T-cell acute lymphoblastic leukemia (CEM and CEM/dCK-) [5]. In these cell lines IC_50_ of troxacitabine was evaluated using standard cytarabine (Ara-C) [94]. Troxacitabine exhibited significantly higher potency against KB, DU-145, and PC-3 cell lines than cytarabine, while retaining comparable potency against HepG2, 2.215, and CEM cell lines (Table 9).

**Table 9:** In vitro anticancer activity of troxacitabine (**5**) compared to cytarabine (Ara-C)

Cell lines	Troxacitabine IC_50_ (µM)	Cytarabine (Ara-C) IC_50_ (µM)	Specific activity (nmol/min/mg)	
			dCyd kinase	dCyd deaminase
KB	0.048 ± 0.021	0.152 ± 0.010	0.093 ± 0.019	5.136 ± 0.321
DU-145	0.024 ± 0.020	0.170 ± 0.035	0.158 ± 0.014	6.122 ± 0.154
PC-3	0.056 ± 0.039	0.200 ± 0.078	0.139 ± 0.010	5.454 ± 0.014
HepG2	0.110 ± 0.050	0.185 ± 0.024	0.131 ± 0.014	0.101 ± 0.018
2.2.15	0.110 ± 0.011	0.145 ± 0.007	0.140 ± 0.020	0.133 ± 0.011
CEM	0.025 ± 0.030	0.030 ± 0.010	0.158 ± 0.038	0.095 ± 0.023
CEM/dCK-	9.250 ± 0.500	15.50 ± 1.000	0.009 ± 0.005	0.090 ± 0.027

However, like cytarabine, troxacitabine was found to be ineffective against CEM/dCK⁻ cell lines lacking deoxycytidine kinase (dCK). It demonstrates that, despite being an unnatural nucleoside, troxacitabine remains dependent on dCK for phosphorylation to convert to the active triphosphate form [5]. Furthermore, all cell lines were also evaluated for levels of dCK and deoxycytidine deaminase (dCD). These analyses revealed a correlation whereby cell lines with high dCD expression were more sensitive to troxacitabine than to cytarabine. Elevated dCD levels reduced the efficacy of cytarabine due to deamination of the drug to its inactive uracil metabolite (Ara-U). In contrast, troxacitabine retained potent activity in cell lines with high dCD expression (KB, DU-145, and PC-3), indicating that troxacitabine is not a substrate for dCD-mediated deamination [5].

Extended kinetic studies of troxacitabine were conducted using purified human dCK and dCD. These studies demonstrated that troxacitabine is efficiently phosphorylated by dCK, with a *K*_*m*_ comparable to that of the natural substrate 2ʹ-deoxycytidine (dCyd) and is not a substrate for dCD-mediated deamination (Table 10).

**Table 10:** Troxacitabine reactivity with dCK and dCD compared to natural analog dCyd

Substrate	dCK		dCD
	*K*_*m*_ (µM)	Relative V_max_	
dCyd	10.57 ± 2.25	1.00	1.00
Troxacitabine	14.21 ± 2.59	0.22	<0.001

*K*_*m*_: Michaelis Constant, *dCyd* 2′-deoxycytidine, *dCK* deoxycytidine kinase, *dCD* deoxycytidine deaminase

*V*_*max*_: highest possible rate of an enzyme-catalyzed reaction

Notably, troxacitabine’s inability to undergo conversion to an inactive uracil metabolite confers a significant advantage over other approved nucleoside analogs. Mechanism of action studies proved that troxacitabine incorporates into the elongating DNA chain, and due to the lack of 3ʹ-OH, acts as a chain terminator [5]. In vivo, troxacitabine was tested in nude mice bearing HepG2 and DU-145 xenografts. It was found that troxacitabine effectively reduces the tumor growth within 15 days of treatment and was safe at a 50 mg/Kg dose [5]. Additionally, troxacitabine inhibited the growth of colon tumors and prolonged the survival of mice bearing p338 leukemia cells [5]. Troxacitabine has shown broad tumor cytotoxicity and was found effective against human tumor colony-forming unit (HTCFU) [84]. It was further advanced to the phase I/II clinical trial to treat refractory acute myeloid leukemia [95]. However, troxacitabine was later dropped from the clinical trials due to its limited efficacy against various cancers compared to other approved nucleosides. In 2005, troxacitabine was granted orphan drug designation (ODD) by the FDA and the European Medicines Agency (EMA) to treat acute myeloid leukemia (AML) but was withdrawn in 2008 [96]. Currently, to enhance the PK and anticancer profile of troxacitabine, various prodrug strategies are underway, and recently, a phosphoramidate prodrug of troxacitabine was introduced as a clinical candidate for the treatment of liver cancer [91].

## Conclusions

Dioxolane-derived nucleos(t)ides (DDNs) are actively investigated as promising scaffolds in antiviral and anticancer drug discovery and development. These molecules have demonstrated potent therapeutic efficacy against a range of viruses. However, so far, none of these DDNs have received FDA approval for treatment. Currently, POM-L-BHDU-MP has only been advanced for the treatment of shingles, oral, and genital herpes. The replacement of the ribose ring of nucleosides with either the natural (D-form) or the unnatural (L-form) 1,3-dioxolane ring generates altered nucleosides specific to virulent viruses and highly dividing cancer cells. The unique features of DDNs place them in a special niche in medical chemistry for their exploration against emerging viruses and cancers. DDNs, as therapeutics, interact with DNA polymerase and inhibit DNA chain elongation in highly dividing cancerous cells and viruses. Furthermore, a detailed SAR exploration of both D- and L-dioxolane-derived nucleosides remains warranted, particularly through alternative base modification strategies. In future studies, structural modifications at the 2ʹ- and 4ʹ-positions of DDNs may offer significant opportunities for the development of new antiviral agents. This review focuses on the synthesis and preclinical/clinical development of DDNs in the antiviral and anticancer fields and timely highlights two molecules, POM-L-BHDU-MP and L-HDVD, with therapeutic potential for treating infections caused by VZV, HSV, EBV, and KSHV. We hope that this report will spark some interest in the synthesis of modified DDNs and the development of new molecules of this class as antiviral and anticancer agents.

## Data Availability

There is no primary research data, code or software included in this manuscript and no new data have been included as part of this review.

## Abbreviations

AZT: Zidovudine (azidothymidine)
ACV: Acyclovir
AML: Acute myeloid leukemia
BVDU: Brivudine
BVU: Bromovinyluracil
BF_3_^.^Et_2_O: Boron trifluoride diethyl etherate
BCl_3_: Boron trichloride
CDV: Cidofovir
CCl_4_: Carbon tetrachloride
DDNs: 1,3-dioxolane-derived nucleos(t)ides
DAPD: Amdoxovir
DXG: D-Dioxolane guanine
DMAP: 4-Dimethylaminopyridine
DPD: Dihydropyrimidine dehydrogenase
DIC: *N*,*N*′-diisopropylcarbodiimide
dCD: Deoxycytidine deaminase
dCK: Deoxycytidine kinase
dCyd: 2ʹ-Deoxycytidine
dGTP: 2ʹ-deoxyguanosine triphosphate
dNTPs: Deoxyribonucleoside triphosphates
dThd: Deoxythymidine
EBV: Epstein–Barr virus
EMA: European Medicines Agency
Et_3_N: Triethylamine
5-FU: 5-Fluorouracil
FCV: Famciclovir
HFFs: Human foreskin fibroblasts
HSV-1: Herpes simplex virus 1
HIV: Human immunodeficiency virus
HMDS: Hexamethyldisilazane
HCMV: Human cytomegalovirus
IMP: Inosine monophosphate dehydrogenase
KSHV: Kaposi’s sarcoma-associated herpesvirus
LiOH: Lithium hydroxide
LTBA: Tri-*tert*-butoxyaluminum hydride
MPA: Mycophenolic acid
Mn(OAc)_2_: Manganese(II) acetate
MHV-68: Murine herpesvirus 68
MDR: Multidrug-resistant
NRTI: Nucleoside reverse transcriptase inhibitor
NMP: Nucleoside monophosphates
NMI: *N*-methylimidazole
NaN_3_: Sodium azide
NaIO_4_: Sodium borohydride
NaOCl: Sodium hypochlorite
[(-)-OddC]: Troxacitabine
ODD: Orphan drug designation
PBMC: Peripheral blood mononuclear cells
Pb(OAc)_4_: Lead (IV) acetate
Pd(OH)_2_: Palladium hydroxide
*p*-TsOH: *p*-Toluenesulfonic acid
Pd(Ph_3_P)_2_Cl_2_: Bis(triphenylphosphine)palladium(II)dichloride
RBV: Ribavirin
RuO_2_: Ruthenium(IV) oxide
RT: Reverse transcriptase
SARS-CoV: Severe acute respiratory syndrome coronavirus
SOC: Skin organ culture
SI: Selectivity index
TK-1: Thymidine kinase-1
TAMs: Thymidine analog-associated mutations
TMSOTf: Trifluoromethanesulfonate
TBDPSCl: *Tert*-butyldiphenylsilane chloride
TBAF: Tetrabutylammonium fluoride
TMS-Cl: Trimethylsilyl chloride
TPase: Thymidine phosphorylases
VACV: Valacyclovir
WT: Wild-type
